# Development and Characterization of Bioinspired Lipid Raft Nanovesicles for Therapeutic Applications

**DOI:** 10.1021/acsami.2c13868

**Published:** 2022-11-30

**Authors:** Lalithasri Ramasubramanian, Harsha Jyothi, Leora Goldbloom-Helzner, Brandon M. Light, Priyadarsini Kumar, Randy P. Carney, Diana L. Farmer, Aijun Wang

**Affiliations:** † Department of Surgery, School of Medicine, University of California-Davis, Sacramento, California 95817, United States; ‡ Institute for Pediatric Regenerative Medicine, Shriners Hospitals for Children, Sacramento, California 95817, United States; § Department of Biomedical Engineering, University of California-Davis, Davis, California 95616, United States

**Keywords:** lipid rafts, extracellular vesicles, neuroregeneration, angiogenesis, drug delivery

## Abstract

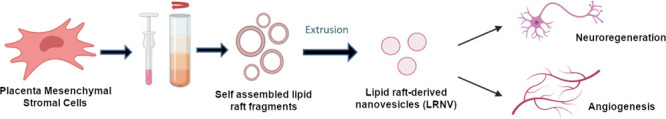

Lipid rafts are highly ordered regions of the plasma membrane enriched in signaling proteins and lipids. Their biological potential is realized in exosomes, a subclass of extracellular vesicles (EVs) that originate from the lipid raft domains. Previous studies have shown that EVs derived from human placental mesenchymal stromal cells (PMSCs) possess strong neuroprotective and angiogenic properties. However, clinical translation of EVs is challenged by very low, impure, and heterogeneous yields. Therefore, in this study, lipid rafts are validated as a functional biomaterial that can recapitulate the exosomal membrane and then be synthesized into biomimetic nanovesicles. Lipidomic and proteomic analyses show that lipid raft isolates retain functional lipids and proteins comparable to PMSC-EV membranes. PMSC-derived lipid raft nanovesicles (LRNVs) are then synthesized at high yields using a facile, extrusion-based methodology. Evaluation of biological properties reveals that LRNVs can promote neurogenesis and angiogenesis through modulation of lipid raft-dependent signaling pathways. A proof-of-concept methodology further shows that LRNVs could be loaded with proteins or other bioactive cargo for greater disease-specific functionalities, thus presenting a novel type of biomimetic nanovesicles that can be leveraged as targeted therapeutics for regenerative medicine.

## 1 Introduction

Lipid rafts are highly ordered subdomains within the cell plasma membrane that result from the thermodynamically driven phase separation of lipids within the membrane bilayer.^1,2^ The lipid raft regions are enriched in cholesterol and glycosphingolipids and present as “raft-like” structures that float among the otherwise fluid plasma membrane. The preferential coalescence of these lipids results in the specific protein recruitment into the lipid raft regions. Many unique protein families, including caveolins,^3,4^ flotillins,^5^ Src family kinases,^6,7^ glycosylphosphatidylinositol (GPI)-anchored proteins,^8^ and growth factor receptors,^9−11^ are selectively partitioned into the lipid raft regions through different mechanisms of raft association. The lipid and protein compositional differences within the lipid raft contribute quite significantly to its functional role in intercellular and intracellular communication via lipid–lipid, lipid–protein, or protein–protein interactions. As a result, lipid rafts are known to be highly involved in the modulation of cell adhesion, migration, and signal transduction.^1,2,12^

Recently, the biological potential of lipid rafts has been highlighted by their role in extracellular vesicle (EV) biogenesis and function. EVs are small nanovesicles secreted by all types of cells and are ubiquitously present in biological fluids. Specifically, exosomes, a subclass of EVs, are derived from the invagination of the vesicles through the lipid raft domains.^13^ As a result of this fusion, the exosomal outer membrane retains many of the functional lipids and surface proteins that are also present in the lipid rafts.^1^ Due to their bioactive membrane and functional cargo, exosomes, and the larger grouping of EVs and associated particles, can modulate important biological processes, including cell proliferation,^14,15^ angiogenesis,^16−18^ immunomodulation,^19,20^ and neuroprotection,^19,21^ thus making them attractive nanotherapeutics for regenerative medicine. However, the actual clinical translation of exosomes has been challenged by the difficulties in EV isolation, purification, and standardization.^22,23^ Even with the use of current gold-standard techniques, EV yield is very low and results in a heterogeneous population of vesicular bodies, which includes other types of vesicles such as microvesicles and apoptotic bodies. Therefore, this limits the large-scale manufacture and standardization of therapeutically significant EV doses. One method by which these challenges can be overcome is to engineer biomimetic nanovesicles that are homogeneous and can be synthesized on a large scale while retaining functional therapeutic properties. EV structures can be broadly grouped into two categories, the membrane shell and the internal cargo, and successful biomimetics need to replicate both constituents. The focus of this current study will be to solely recapitulate the exosome-specific membrane by establishing a biomaterial source that can express similar biomolecules found within native exosome membranes. Lipid rafts are a promising choice of a biomaterial due to their role in exosome biogenesis.^13,24^ Here, we hypothesized that lipid rafts can function as bioactive biomaterials comparable to exosome membranes and from which lipid raft-derived nanovesicles (LRNVs) can be synthesized.

In this study, we use early gestation human placenta-derived mesenchymal stromal cells as a model cell line for lipid raft derivation and LRNV synthesis. We have previously shown that EVs derived from early gestation human placenta-derived mesenchymal stromal cells (PMSCs) have significant neuroprotective^19,21^ and angiogenic properties,^25^ making them a promising multifunctional therapeutic for regenerative medicine. Thus, any LRNVs engineered from PMSCs should exhibit similar regenerative potential. Here, we show that detergent-resistant membrane fractions can be isolated from PMSCs using density-based gradient ultracentrifugation and identified as lipid rafts. The biochemical compositions, namely the lipid and protein constituents, were studied with comparative lipidomic and proteomic analyses to assess the degree of similarity to native PMSC EVs. Lipid rafts were then engineered into nanovesicles using an extrusion-based method, and the resulting LRNVs demonstrated *in vitro* neurogenic and angiogenic effects. As EVs are known to carry a variety of bioactive cargo that augment their biological functions, we also sought to show that LRNVs can also act as drug carriers of various biomolecules. Therefore, we aimed to load a proof-of-concept protein within the LRNVs to further demonstrate the versatility of the LRNVs as a bioinspired nanotherapeutic for a wide range of clinical applications.

## 2 Results

### 2.1 PMSC-Derived Lipid Rafts Can Be Isolated Using Density-Based Gradient Ultracentrifugation and Have EV-like Characteristics

**Figure 1:**
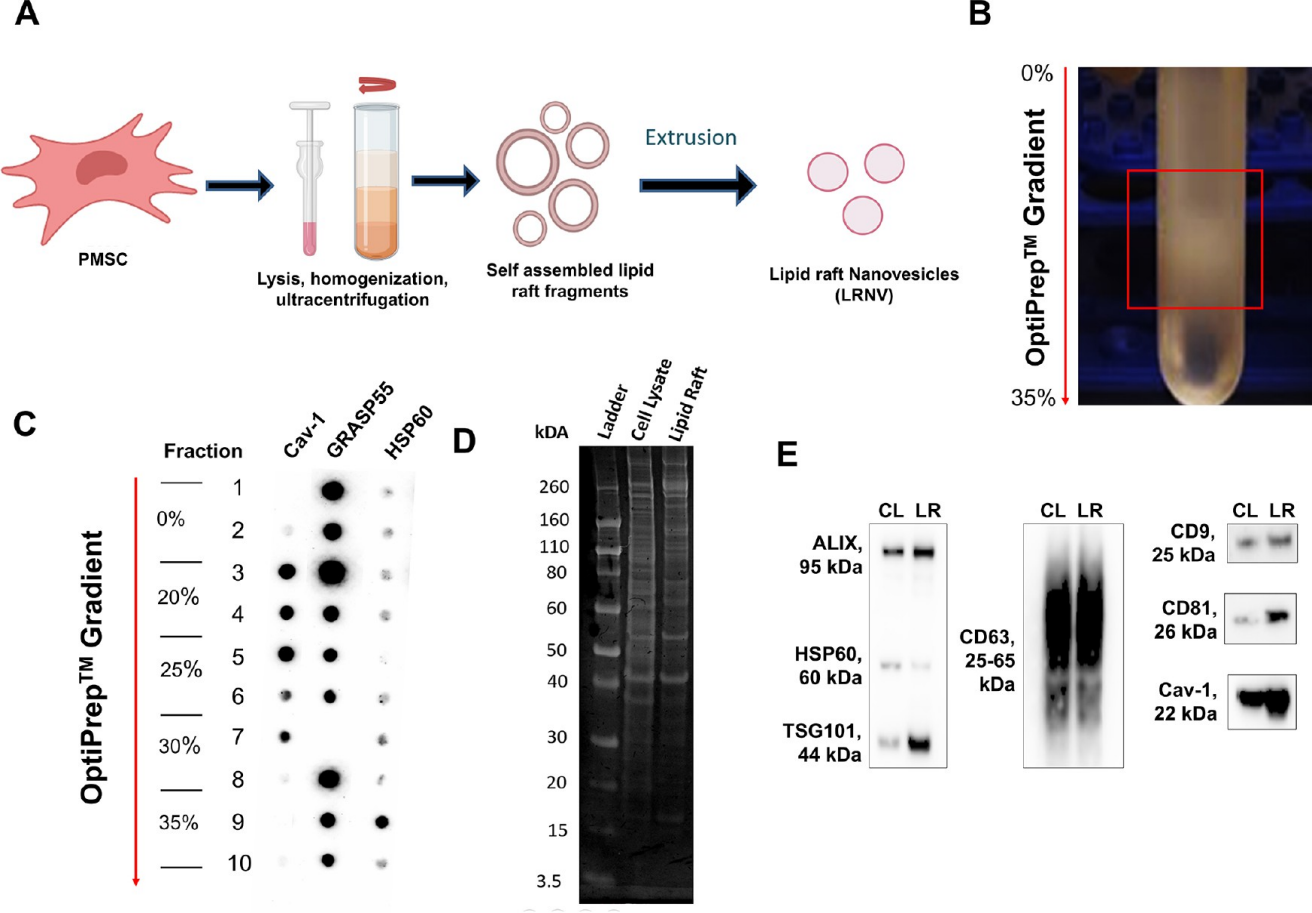
Isolation and characterization of PMSC lipid rafts and LRNVs. (A) Overall schematic of lipid raft isolation and synthesis of LRNVs. (B) Representative image of OptiPrep gradient ultracentrifugation. Box highlights the collection of lipid raft fragments at the 20–30% fraction. (C) Dot plot analysis of caveolin-1 (cav-1), GRASP55, and HSP60 expression at different fractions (1 at 0%, 10 at 35%) of the gradient. (D) Five micrograms of whole cell lysates and lipid raft isolates from the same cell line was resolved by SDS-PAGE, and proteins were visualized using Imperial Protein gel stain. (E) Representative Western blot of whole cell lysates and lipid raft isolates probed for common EV markers (ALIX, TSG101, CD9, CD63), lipid raft marker cav-1, and mitochondrial marker HSP60 as the negative control.

Lipid rafts were isolated from PMSCs, which we have shown to have high regenerative potential, using an Optiprep density gradient ultracentrifugation method (Figure 1A). Following the gradient ultracentrifugation, a dense band was seen in the 25–30% layers (Figure 1B). Dot plot analysis was conducted to assess the expression of caveolin-1 (cav-1, lipid raft marker), Golgi reassembly-stacking protein of 55 kDa (GRASP55, Golgi membrane marker), and heat shock protein 60 (HSP60, mitochondrial membrane marker) in each fraction. Fractions within 20–25–30% gradient layers (ρ = 1.11–1.19 g/mL) were found to have an enriched expression of cav-1 and a depleted expression of GRASP55 and HSP60, indicating subcellular localization of lipid rafts within these fractions (Figure 1C). The lipid raft-enriched fractions were pooled, and sodium dodecyl-sulfate polyacrylamide gel electrophoresis (SDS-PAGE) was used to visualize total protein expression (Figure 1D). Qualitative comparison between equal amounts of whole cell lysates and the lipid raft isolate from the same cell line showed that many proteins were retained and, at times, enriched in the lipid rafts. However, there was also a reduction in the intensity of many protein bands, likely the cytosolic proteins that were removed during the isolation process. To confirm, the lipid raft sample was further subjected to more specific Western blot analysis. Samples were probed for cav-1 and HSP60 in addition to commonly accepted EV markers ALIX, TSG101, CD9, CD63, and CD81 (Figure 1E).^26^ In comparison to cell lysate controls, lipid raft isolates demonstrated a high expression of caveolin-1 but a low expression of HSP60, confirming the enriched lipid raft composition of the final isolate. All probed EV markers were retained in the lipid raft isolates, and in the case of TSG101 and CD81, they were found to be enriched compared to the whole cell lysates.

### 2.2 Lipidome of PMSC Lipid Rafts Is More Similar to EVs than to Source Cells but Still Present with Some Unique Differences

**Figure 2:**
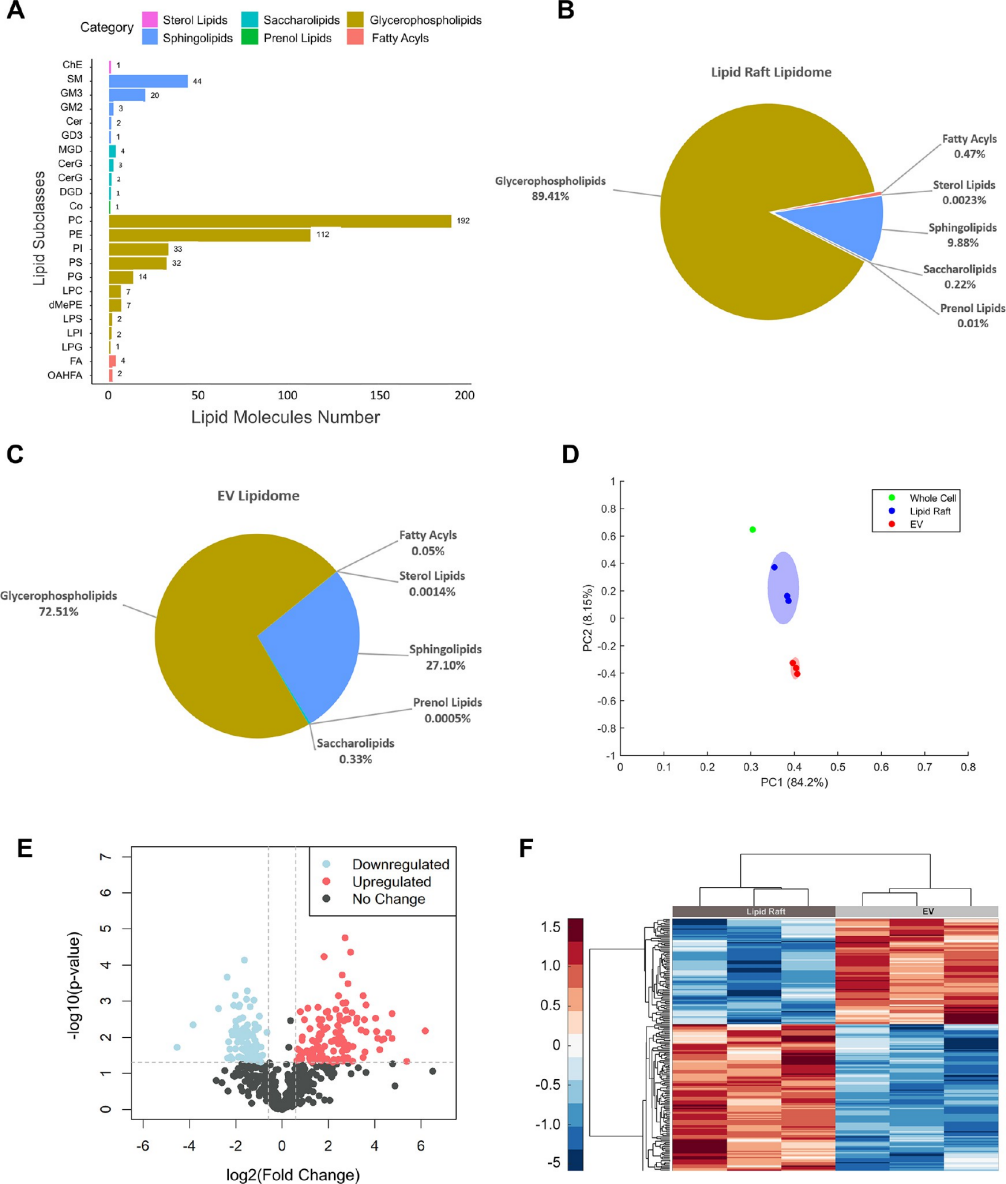

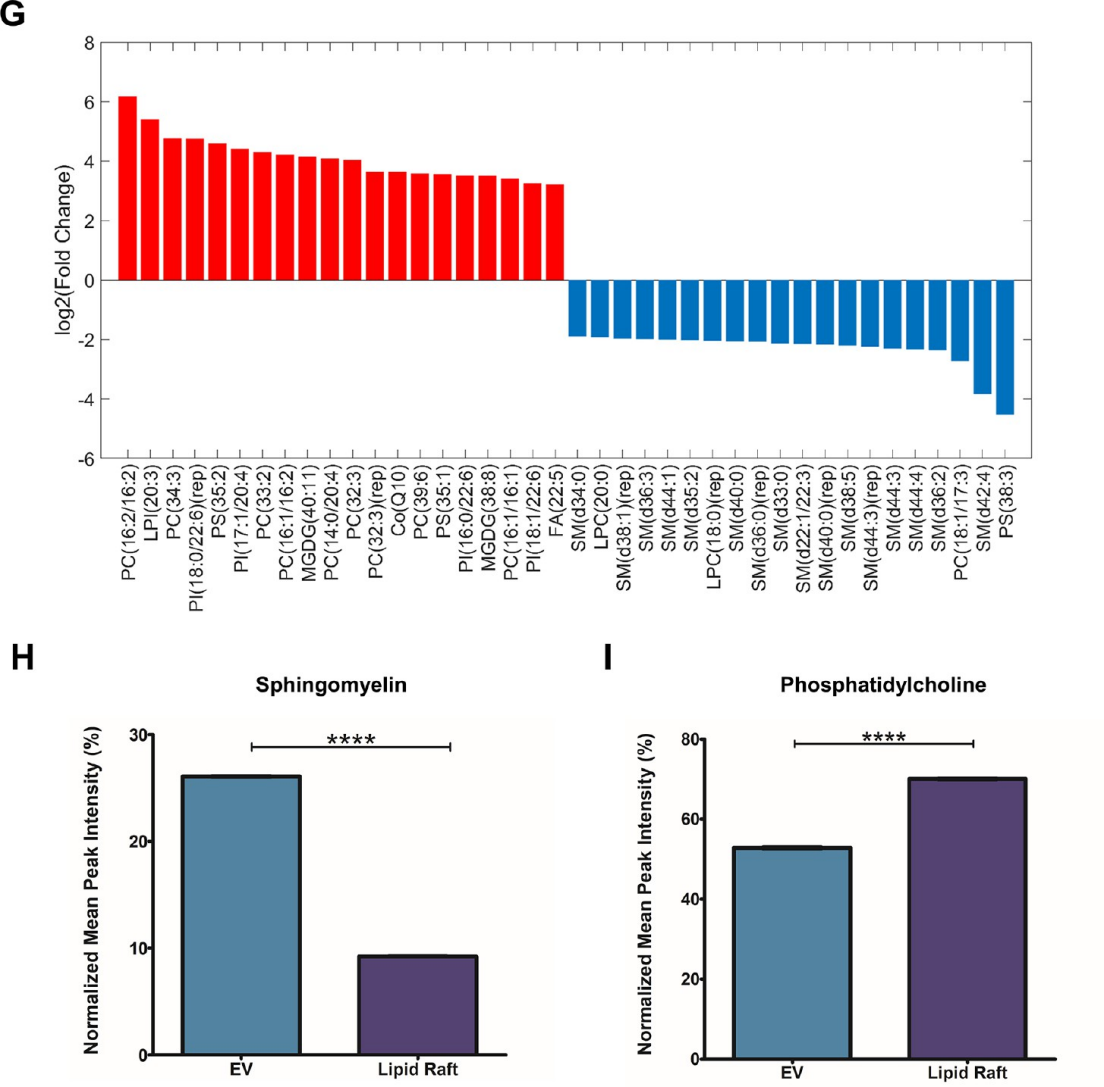
Lipidomic analysis of PMSC-derived EVs and lipid rafts using LC-MS/MS. (A) A total of 490 unique lipid molecules divided into six lipid classes were identified in both EV and lipid raft samples. The lipid composition of (B) lipid rafts and (C) EVs were compared based on the normalized abundance of lipids in each lipid class. (D) First and second principal component scores for lipid ions detected in whole cell lysates (*n* = 1 cell line), EVs (*n* = 3 cell lines), and lipid rafts (*n* = 3 cell lines). Shapes represent the cluster membership at a 95% confidence interval. (E) The volcano plot depicts the 207 differentially expressed lipids in lipid rafts compared to EVs, defined as *p*-value < 0.05 (Student’s *t*-test) and a fold change >1.5. (F) Hierarchical cluster analysis with Euclidean distance measurement was used to generate a heatmap of distinct clusters of enriched lipid molecules in EVs or lipid rafts. (G) Differentially expressed lipid species in lipid raft samples compared to EVs were quantified by log 2(Fold Change), and the top 20 upregulated and downregulated lipid species are shown. The complete list of differentially expressed lipid (DEL) species can be found in the Supporting Information. Major types of differentially expressed lipids include (H) sphingomyelin and (I) phosphatidylcholine. *** *p* < 0.001 by Student’s *t*-test.

To determine whether lipid rafts are a feasible EV-mimicking biomaterial source, we first compared the lipidome profiles between native PMSC EVs and lipid rafts from the same donor cell lines for matched analysis. PMSC EVs were isolated through differential ultracentrifugation. Samples were confirmed to be EVs through protein expression, size, morphology, and ζ-potential characterization as recommended by the minimum experimental guidelines set by the International Society of Extracellular Vesicles (Figure S1).^26^ Liquid chromatography with tandem mass spectrometry (LC-MS/MS) analysis was conducted to identify lipid molecules across all samples. In total, 490 unique lipid groups from six overall lipid categories were identified in both EV and lipid raft lipidomes (Figure 2A). Relative abundance of lipids in each sample was quantified and averaged across three replicates to obtain an overall lipidome profile for EVs and lipid rafts. Both EVs and lipid rafts were predominantly composed of glycerophospholipids and sphingolipids with some minor presence of fatty acyl lipids and saccharolipids (Figure 2B,C). However, the percent composition of glycerophospholipids and the sphingolipids across two groups differed. Lipid rafts had a greater percentage of glycerophospholipids than the EVs (89.4 vs 72.5%) while EVs presented with a higher proportion of sphingolipids (9.88 vs 27.1%). Compared to the whole cell lipidome, both lipid rafts and EVs expressed an enrichment in sphingolipids and saccharolipids but a decrease in fatty acyls and glycerophospholipids (Figure S2). To investigate whether these differences were significant, we first performed multivariate analysis to compare the variation in the overall lipidome profile between whole cells, lipid rafts, and EVs. Principal component analysis showed a clear separation between all three groups with replicates of each group clustering together and suggesting a certain level of unique features among each group (Figure 2D). Interestingly, the whole cell and EV had the greatest separation with the lipid rafts in between, highlighting the probability of lipid raft samples resembling both whole cells and EVs. Next, any differentially expressed lipids between EVs and lipid rafts were investigated using univariate analysis. A volcano plot was constructed by applying FC > 1.5 and a significance of *p* < 0.05 based on t-test analysis (Figure 1E). This revealed a total of 207 lipid ions with significant changes in expression with downregulation of 86 (13.9%) lipid ions and upregulation of 121 (19.5%) lipid ions in lipid rafts compared to EVs and 412 lipid ions (66.6%) with no change. We applied hierarchical cluster analysis to visualize the grouping of relatively expressed lipids across individual replicates of both sample groups (Figure 2F). Unsupervised clustering analysis revealed a good level of homogeneity within each sample group as all replicates clustered together, and a pattern of upregulation and downregulation of lipid types was observed across the two groups of samples. To better understand the specific lipids that were differentially expressed, the fold change of each differentially expressed lipid ion was quantified (Figure 2G). Differential expression of grouped lipid species was also compared (Figure S3). The predominant differences were due to varied expression within the glycerophospholipid and sphingolipid subclasses with lipid rafts displaying a significantly higher expression of phosphatidylcholine and lower expression of sphingomyelin compared to EVs (Figure 2H,I).

### 2.3 Proteome of Lipid Rafts Display High Biological Potential Even with Slight Donor Variability

**Figure 3:**
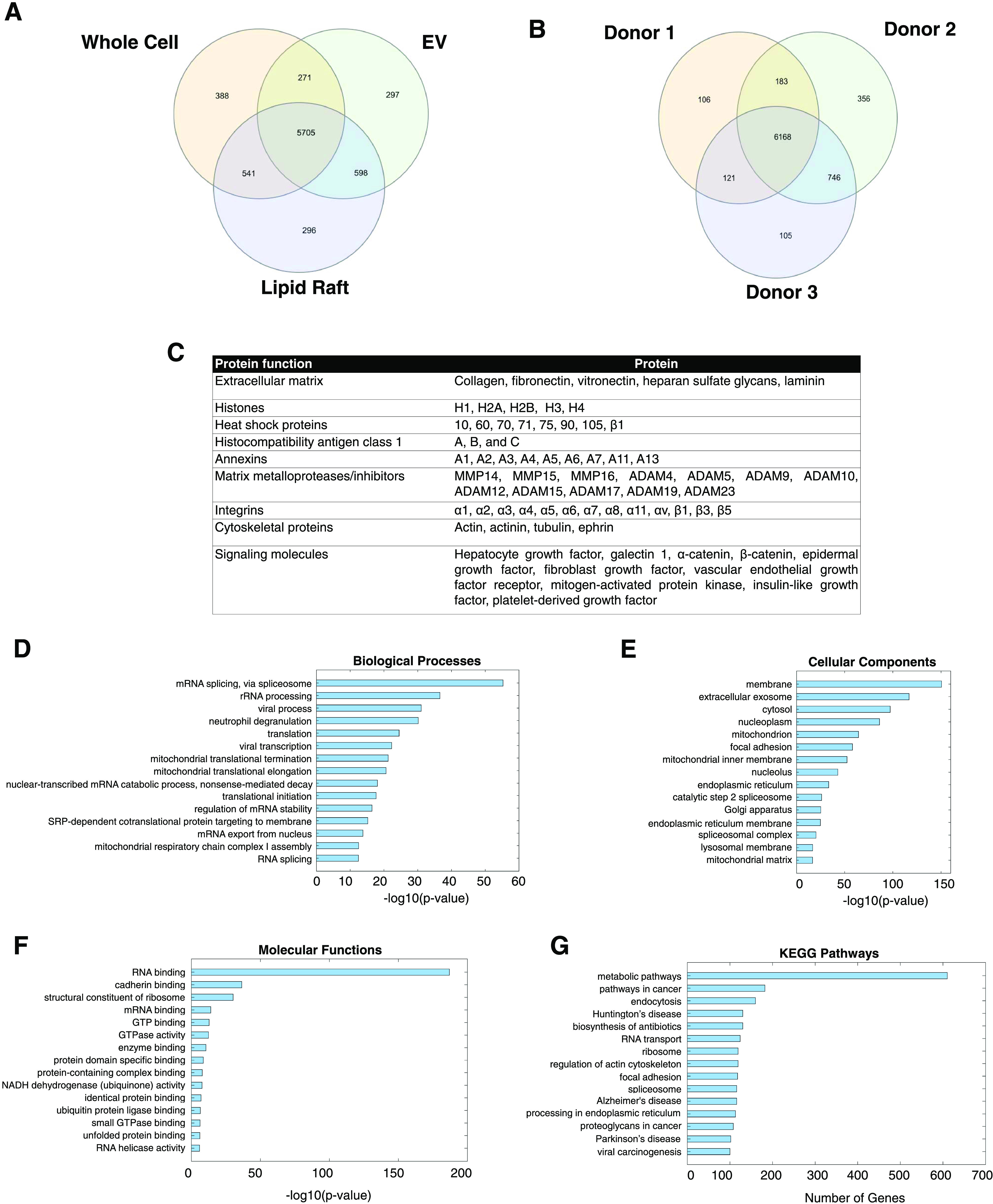
Proteomic analysis of PMSC-derived lipid rafts. Number of common proteins was compared between (A) whole cell lysates, EVs, and lipid rafts from the same line and (B) between lipid raft samples from three different cell lines. (C) The 6168 conserved proteins in lipid rafts from all donors were analyzed for vital structural and signaling proteins. Gene ontology searches for (D) biological processes, (E) cellular components, (F) molecular functions, and (G) KEGG pathway analysis were conducted using FunRich software and DAVID.

The protein composition of the lipid rafts was characterized by proteomic analysis using tandem mass spectrometry. Before analysis, bovine serum albumin (BSA) and keratin (contamination from sample processing procedures) were manually removed from the dataset. Proteomes of whole cells, EVs, and lipid rafts from the same cell line were compared (Figure 3A).^27^ In total, 5705 proteins (70.5%) were conserved among all three groups. When the proteomic similarity between all permutations of the groups were compared, EVs/lipid rafts had the most similar proteomic profile at 81.8%, followed by lipid rafts/whole cells at 80.1% and EVs/whole cells at 76.6%. Next, proteomes of lipid rafts from all three cell lines were compared to evaluate any donor-associated variability and heterogeneity (Figure 3B). In total, 6168 proteins, or 79.2%, were conserved across all donors. Analysis of the common proteins identified the presence of many growth factors, signaling proteins, and integrins (Figure 3C).^19^ Conserved proteins in the lipid rafts were then evaluated for functional enrichment and network analysis with gene ontology searches using FunRich software and Database for Annotation, Visualization, and Integrated Discovery (DAVID). Lipid raft proteins were found to be significantly involved in the RNA processing pathway (Figure 3D), while cellular component analysis revealed highest significant enrichment in membrane and extracellular exosome (EV) proteins (Figure 3E). Pathway analyses for molecular functions and KEGG pathways revealed most significant involvement in RNA binding (Figure 3F) and metabolic pathways (Figure 3G). Complete datasets for all GO analysis and KEGG pathway analysis can be found in the Supporting Information.

### 2.4 Synthesis and Characterization of LRNVs

**Figure 4:**
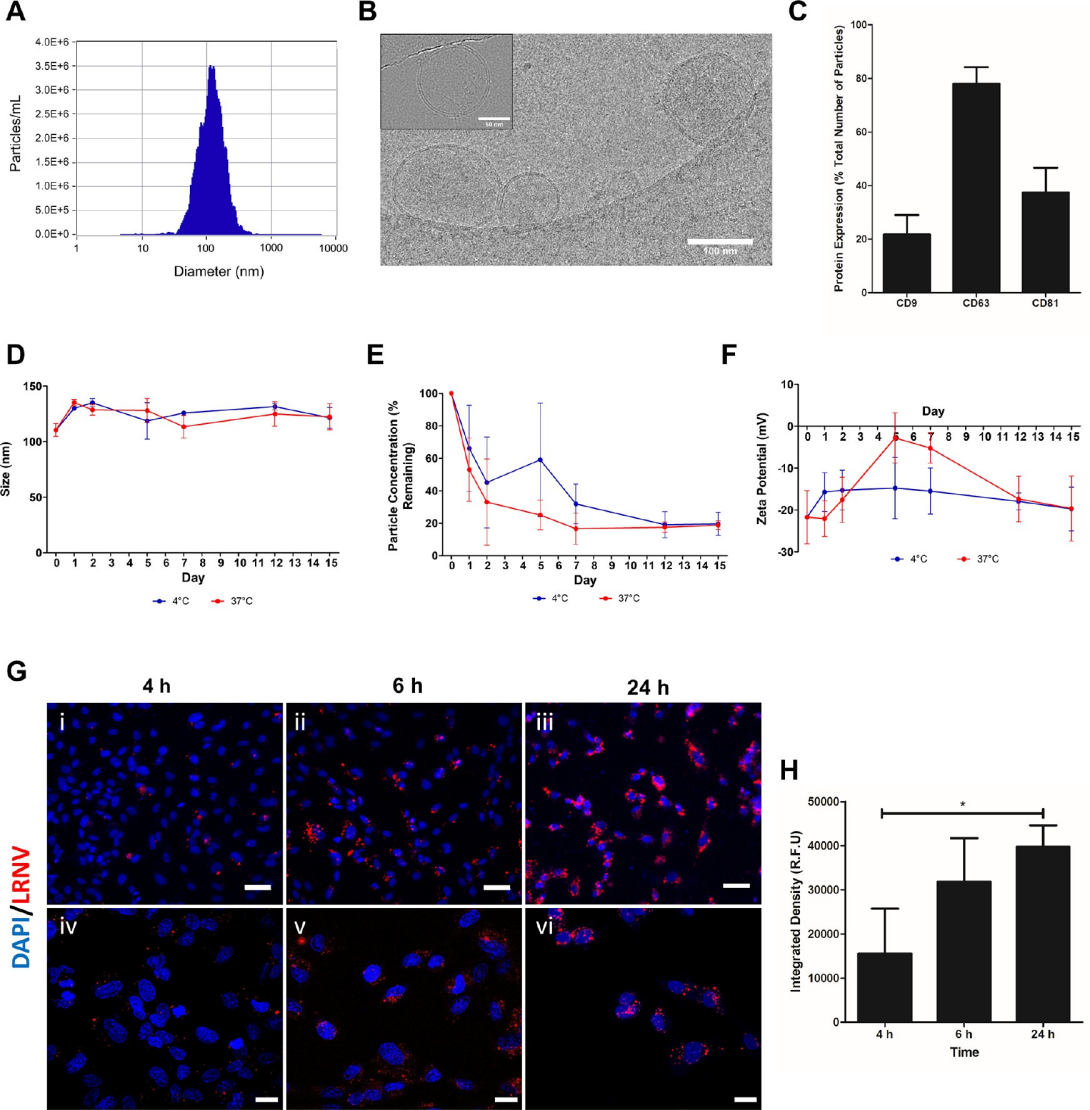
Lipid rafts were extruded through polycarbonate membranes to generate LRNVs. (A) NTA measurements of LRNV size (diameter). (B) Representative cryoEM image of LRNV particles; scale bar: 100 nm. Inset showing higher resolution image of a single LRNV particle; scale bar: 50 nm. (C) Following synthesis, LRNVs were probed for surface expressions of tetraspanins CD9, CD63, and CD81 using ExoView analysis to ensure retention of surface markers (*n* = 3). Hydrodynamic stability of LRNVs was measured over 15 days at 4 °C in water or 37 °C in phosphate buffered saline (PBS) by assessing changes in the (D) size, (E) concentration, and (F) ζ-potential (*n* = 3, ±S.D). (G) Fluorescent images of human umbilical vein endothelial cells (HUVECs) after incubation with DiD-labeled LRNVs (red). Nuclei were visualized with 4′,6-diamidino-2-phenylindole (DAPI) (blue). Cells were incubated with DiD-LRNVs for 4, 6, or 24 h. (i–iii) Images at 20× magnification; scale bar: 50 μm, and (iv–vi) 60× magnification; scale bar: 20 μm. (H) LRNV uptake at all three timepoints was semiquantitatively measured using relative fluorescent intensity (*n* = 3).

Isolated lipid raft fractions were extruded through a 200 nm polycarbonate filter to assemble lipid raft-derived nanovesicles (LRNVs). Compared to unextruded lipid raft isolates, LRNVs demonstrated more uniform size distributions and larger average particle sizes (Figure S4A). Nanoparticle tracking analysis (NTA) revealed a mean diameter of 122.6 ± 5.27 nm (Figure 4A). Morphological analysis by cryoEM depicted a spherical structure with a clear bilayer, indicating the successful synthesis of vesicular structures (Figure 4B). From 1 × 10^6^ cells, approximately 1.18 × 10^11^ ± 4.39 × 10^10^ LRNVs were synthesized, a much higher yield than matched EV yields (Figure S1D). Following synthesis, LRNVs were subjected to ExoView analysis to evaluate the surface expression of CD9, CD63, and CD81 (Figure 4C). ExoView is a multiplexed, immunocapture-based system that can detect the expression of surface proteins on nanovesicles based on fluorescent antibody staining. The presence of all three tetraspanins was confirmed on the LRNVs, with 21.8% particles expressing CD9, 80.0% expressing CD63, and 37.5% expressing CD81. Interestingly, the distribution of the surface proteins on the LRNVs was similar to what was previously observed on the PMSC EVs, further highlighting their membrane surface similarities (Figure S1). We then confirmed the stability of the LRNVs over 15 days under storage conditions (i.e., 4 °C) or at physiological conditions (i.e., 37 °C). The hydrodynamic diameter of the LRNVs did not significantly change over time at either temperature condition, which indicated a certain level of particle stability in aqueous systems (Figure 4D). However, there was a noticeable decrease in particle concentration over time under both conditions, which may indicate that LRNVs are degrading over time (Figures 4E and S4B). This was further bolstered by the trend of ζ-potential measurements over time (Figure 4F). LRNVs exhibited an initial net negative charge due to the anionic nature of proteins expressed on the particle surface.^28^ Over time, however, an increase in the ζ-potential was observed, particularly at 37 °C conditions, before returning to a more negative plateau at the end of 15 days.

To effect a biological change in target cells, LRNVs must be efficiently uptaken by cells. Thus, HUVECs were incubated with DiD-labeled LRNVs for 4, 6, or 24 h to visualize particle uptake into cells (Figure 4G). DiD-only samples were also prepared and incubated with HUVECs as a control group to account for background fluorescence from uptake of any DiD micelles (Figure S5). Fluorescence microscopy revealed an accumulation of LRNVs over time, indicating a time-dependent uptake mechanism (Figure 4H).

### 2.5 LRNVs Retain Signal Transduction Functions That Are Characteristic of Lipid Rafts

**Figure 5:**
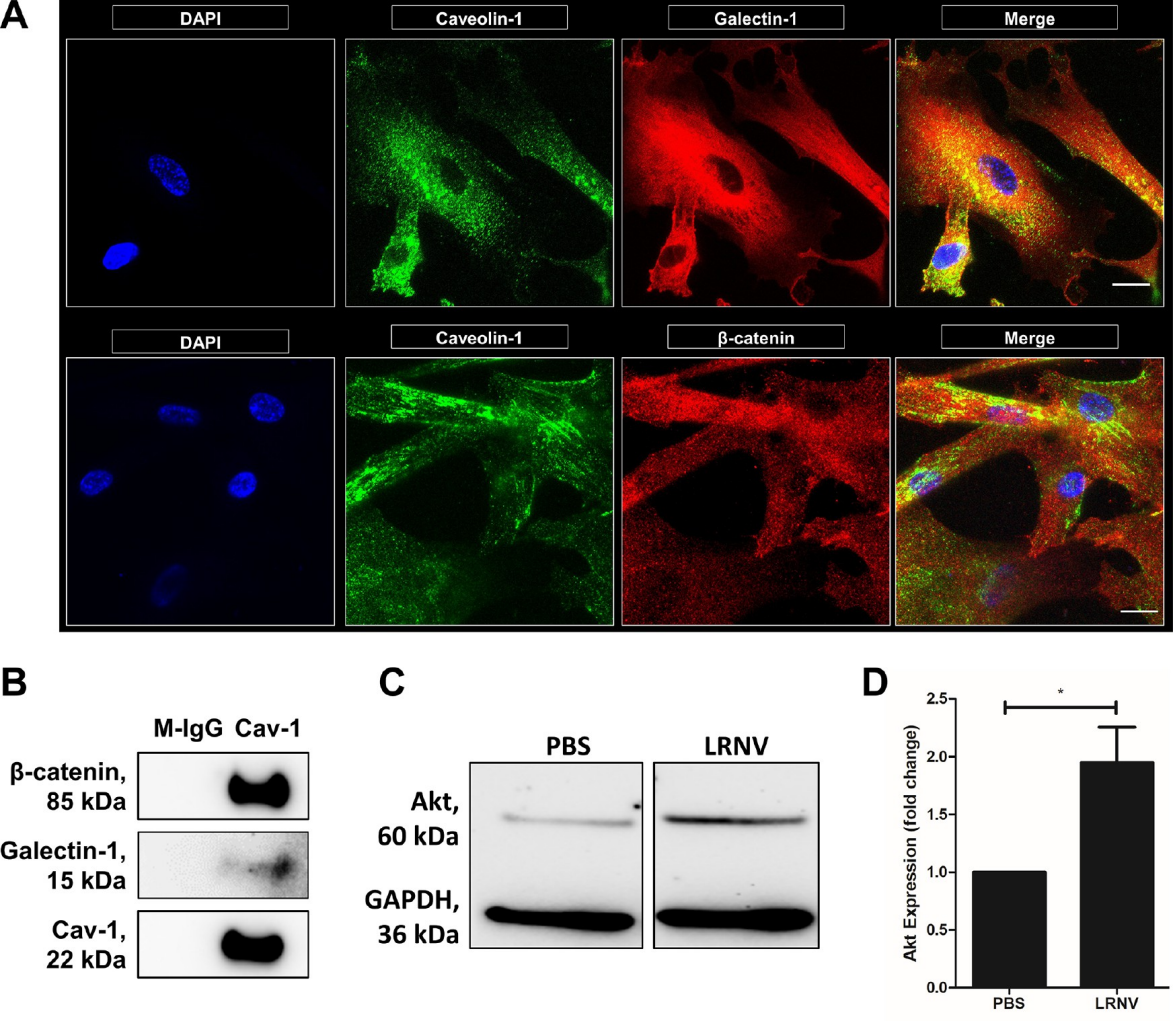
Cell signaling properties of lipid rafts and LRNVs. (A) PMSCs were immunolabeled for caveolin-1 (green) and galectin-1 (top) or β-catenin (bottom) (red) to visualize colocalization of bioactive signaling proteins within the lipid raft microdomains. Scale bar: 20 μm. (B) Colocalization was further confirmed with co-immunoprecipitation of β-catenin or galectin-1 with caveolin-1. Mouse-IgG (M-IgG) was used as an antibody control for caveolin-1. (C) HUVECs were treated with LRNVs for 48 h and assessed for Akt expression using Western blotting. Glyceraldehyde-3-phosphate dehydrogenase (GAPDH) was used as a loading control. (D) Akt expression was quantified after GAPDH normalization (*n* = 3 donor cell banks and repeated with three independent experiments). *p < 0.05 versus untreated control using a one-sample *t*-test.

Lipid rafts are decorated with highly functional proteins and lipids that facilitate activation of many signaling processes upon receptor-mediated interactions.^1,2^ We hypothesized that LRNVs, due to their lipid raft composition, can function by activating vital signaling pathways. We specifically chose to investigate galectin-1 and β-catenin due to their presence on native PMSC EVs (Figure S6A).^19^ Galectin-1 has known neuroprotective and immunomodulatory properties, while β-catenin was selected due to its well-established role as a signal transducer in the canonical Wnt signaling pathway that has implications for an early neural differentiation process of neural progenitor cells and for endothelial cell proliferation, migration, and survival.^29−32^ First, we confirmed the colocalization of signaling proteins galectin-1 and β-catenin with caveolin-1 by immunocytochemistry (Figure 5A). IgG controls were performed to account for nonspecific staining (Figure S6B). To further confirm that both proteins are membrane-bound within the lipid raft regions, we performed a co-immunoprecipitation. Caveolin-1 was immunoprecipitated from lipid raft isolates, and immunoblotting confirmed that both galectin-1 and β-catenin were co-immunoprecipitated with caveolin-1 (Figure 5B). To determine whether LRNVs retained the signaling properties of lipid rafts, we examined the ability of LRNVs to upregulate the Akt/PI3K signaling pathway, which is widely associated with cell growth and metabolism in both neurogenesis and angiogenesis.^33−36^ Western blot analysis of HUVEC lysates revealed an almost 2-fold increased Akt expression in cells treated with LRNVs compared to the untreated control (Figure 5C,D). These results indicate that LRNVs retain key signal transduction functions that are characteristic of lipid rafts.

### 2.6 LRNVs Exhibit Neurogenic and Neuroprotective Functions

**Figure 6:**
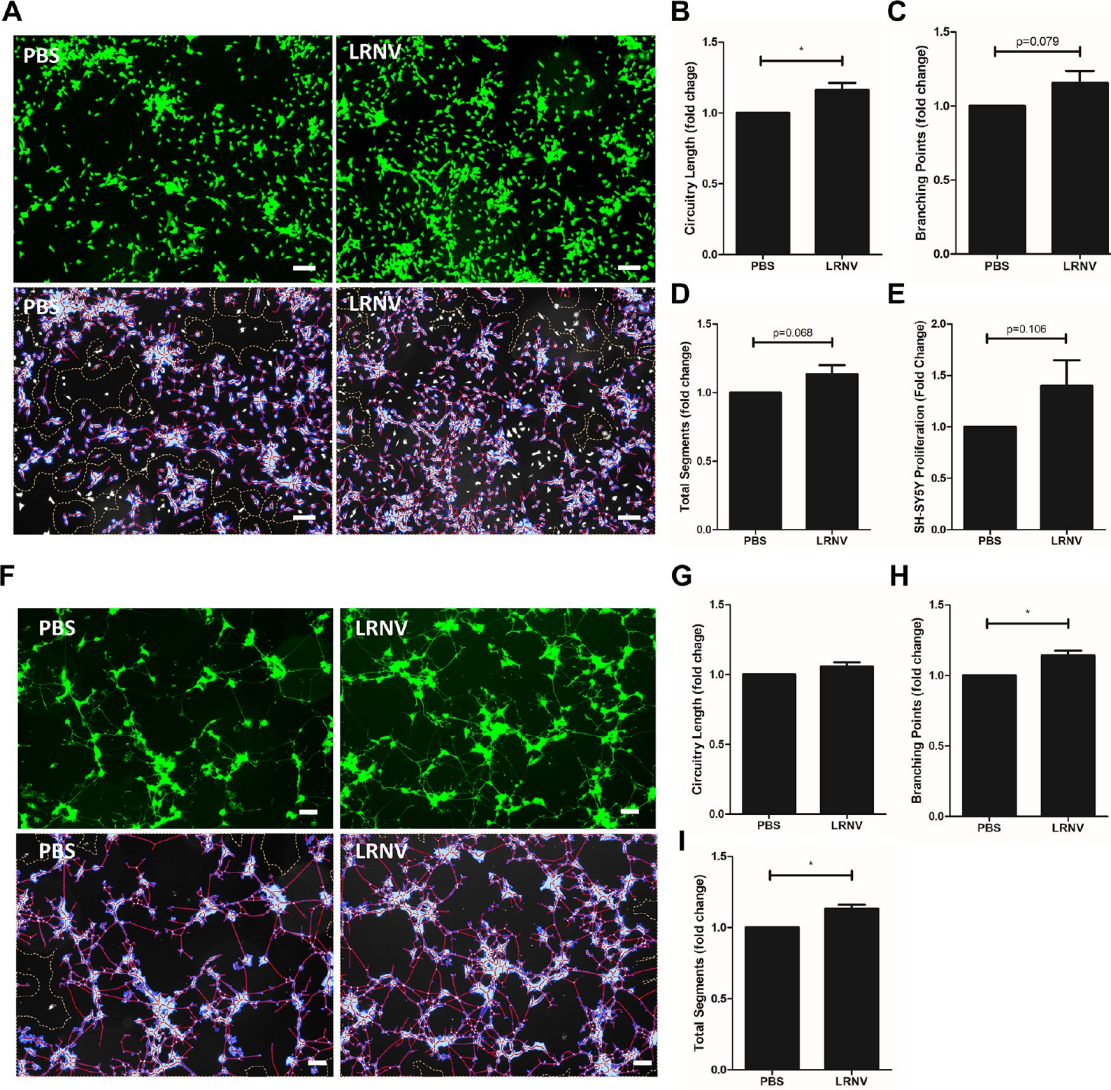
Neuroregenerative properties of LRNVs by direct coculture. (A–E) LRNVs were added directly to SH-SY5Y cells and incubated for 48 h for a neurogenesis model. Repeated *n* = 3 times in triplicate. (A) Top: calcein AM staining, bottom: Wimasis WimNeuron image analysis of representative images of cells in the presence or absence of LRNVs. Scale bar: 100 μm. WimNeuron quantification of (B) circuitry length (px), (C) branching points, and (D) total segments length (px). (E) The effect of LRNVs on SH-SY5Y proliferation was assessed with an MTS assay. Repeated 5 times in triplicate. (F–I) Neurorescue effects of LRNVs were assessed using a neuroprotection model. Apoptosis was induced in SH-SY5Y cells with staurosporine, and cells were then treated with or without LRNVs. Repeated *n* = 4 times in triplicate (F) Top: calcein AM staining, bottom: Wimasis WimNeuron image analysis of representative images of cells with or without LRNV treatment. Scale bar: 100 μm. Images were quantified for (G) circuitry length (px), (H) branching points, and (I) total segments length (px). *n* = 3 donor cell banks for all assays. *p < 0.05 versus untreated control using a one-sample *t*-test.

We have previously found PMSC EVs to possess significant neuroprotective effects and therefore further examined the ability of LRNVs to recapitulate similar functions. We first investigated the ability of LRNVs to promote neurite outgrowth in SH-SY5Y cells. LRNV particles were directly cocultured with SH-SY5Y cells for 48 h, and cells treated with an equivalent volume of PBS served as the control. After incubation, cells were stained with calcein-AM and analyzed using Wimasis WimNeuron (Figure 6A) software to quantify the number of neurite circuitry length, branching points, and total segment length using established protocols.^19^ Image quantification showed that there was a trend of increased circuitry length (Figure 6B), branching points (Figure 6C), and total segment length (Figure 6D); however, only the circuitry length reached significance levels. To assess whether LRNVs can also stimulate SH-SY5Y cell proliferation, an MTS assay was performed. Based on preliminary dose studies, 1 × 10^9^ particles/mL were incubated with SH-SY5Y cells for 48 h, after which the MTS reagent was added to assess cell viability. A trend of increased cell proliferation was noted in the presence of the LRNVs (Figure 6E).

While it is important to promote neurogenesis, in cases of traumatic neural injury like acute spinal cord injury, it is also vital to facilitate the recovery of injured neurons. Therefore, to model the ability of LRNVs to rescue injured neurons, we first treated SH-SY5Y cells with 0.5 μM staurosporine for 4 h to induce apoptosis.^19^ LRNV particles (1 × 10^9^ particles/mL) were directly cocultured with apoptotic SH-SY5Y cells for 120 h following which cells were stained with calcein-AM and analyzed using Wimasis WimNeuron (Figure 6F). The neurite circuitry length increased compared to the control though the difference was not statistically significant (Figure 6G). However, LRNV treatment did significantly improve both the number of branching points and total segment length, with a 1.14-fold and 1.13-fold increase, respectively (Figure 6H,I).

### 2.7 LRNVs Promote Pro-angiogenic Processes

**Figure 7:**
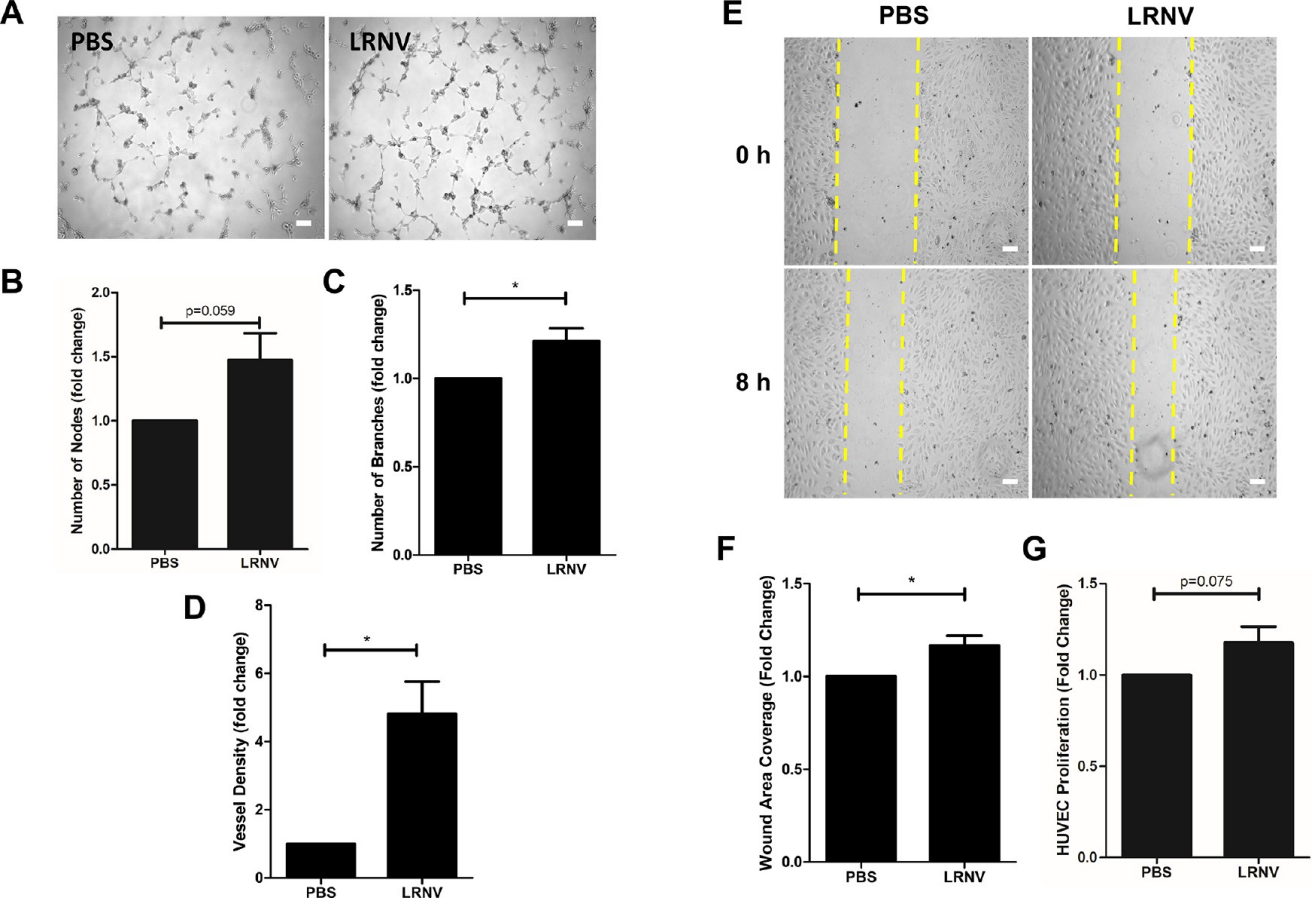
Angiogenic properties of LRNVs. (A) Representative images of HUVEC tube formation in the absence or presence of LRNV after 6 h incubation. Scale bar: 100 μm. ImageJ quantification of (B) number of nodes, (C) number of branches, and (D) vessel density normalized to the control group with no LRNV treatment. (E) Representative images of HUVEC migration at 0 and 8 h incubation with or without LRNV treatment. Scale bar: 100 μm. (F) Quantification of cells migrated into the wound area normalized to no treatment group. (G) MTS assay to assess HUVEC proliferation with LRNV treatment and normalized to the control group without LRNV (*n* = 3 donor cell banks for all assays). Repeated four times in triplicate for tube formation and migration assays and repeated five times in triplicate for an MTS assay. *p < 0.05 versus untreated control using a one-sample *t*-test.

Next, we sought to investigate the pro-angiogenic properties of the LRNVs to further explore their therapeutic potential. One key stage of angiogenesis is tubule formation, where endothelial cells form vascular networks in the presence of an extracellular matrix. Here, HUVECs were seeded on Matrigel and cultured with or without LRNVs for 6 h and then imaged (Figure 7A). Differences in tubulogenesis were measured by ImageJ quantification of the number of tube nodes and number of branches within the vascular network. We found that LRNVs improved the number of nodes, branches, and vessel density compared to untreated control (Figure 7B–D). We next investigated the ability of the LRNVs to improve cell migration. Cell migration is vital to angiogenesis as cell motility is required for vascular remodeling. HUVECs were grown to confluence and then starved in serum-free medium for 16 h to ensure any wound coverage was due solely to cell migration rather than to potential cell proliferation. Following serum starvation, cells were switched to EBM media with 1% BSA and treated with LRNVs. Images were taken at 0 and 8 h, and cell migration was quantified by measuring the remaining wound area (Figure 7E). After 8 h, a significantly smaller remaining wound area was seen with LRNV treatment, suggesting the ability of the LRNVs to promote cell migration (Figure 7F). Finally, HUVEC proliferation was assessed using an MTS assay. HUVECs were incubated with LRNVs for 48 h, and viability was measured. There was a trend of improved cell proliferation with LRNV treatment compared to the control group though it ultimately did not reach significance (Figure 7G).

### 2.8 LRNVs Can Be Loaded with Exogenous Protein Cargo for Enhanced Therapeutic Functions

**Figure 8:**
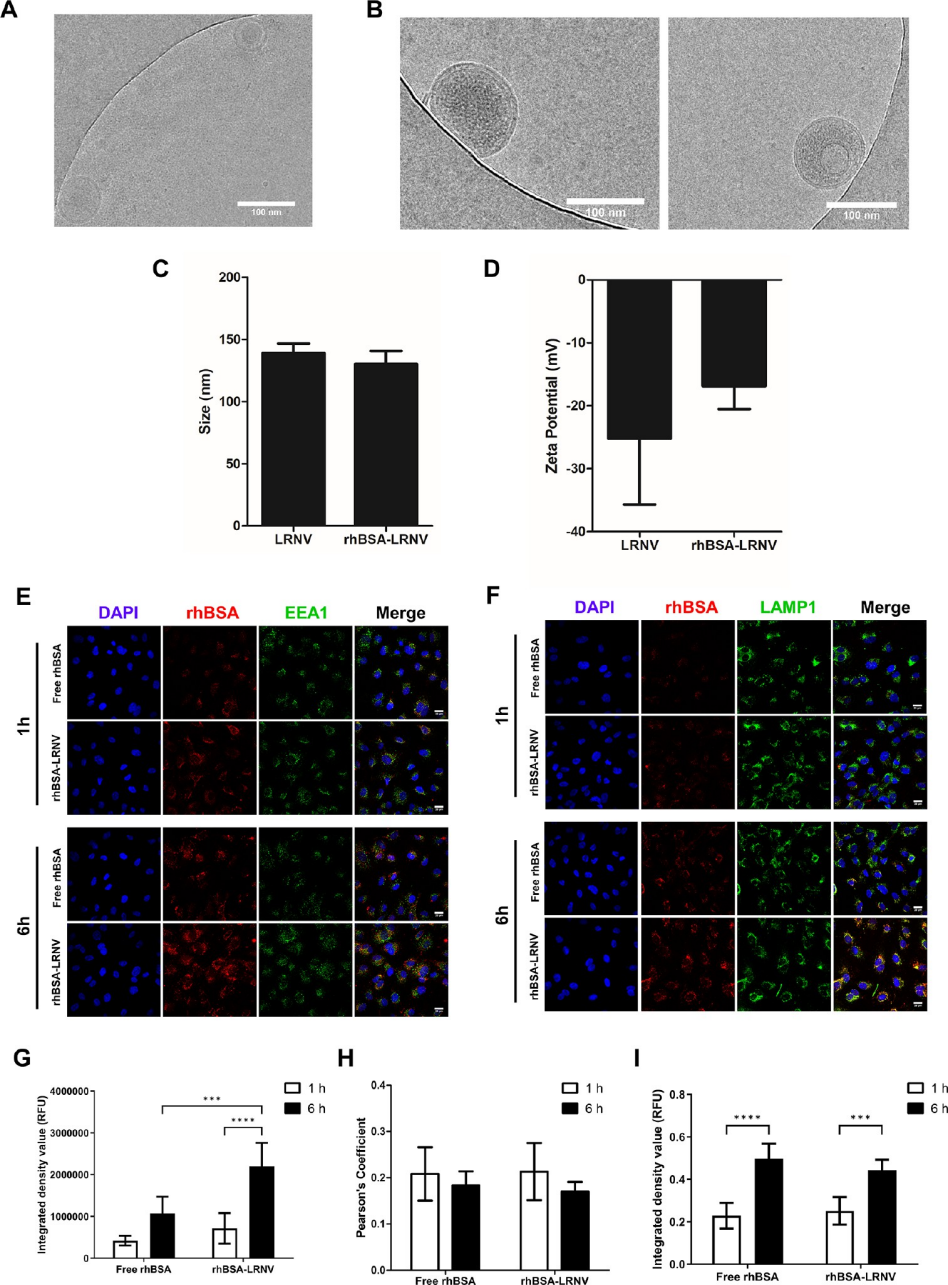
Proof-of-concept cargo loading into LRNVs and cell internalization. Tetramethylrhodamine-conjugated bovine serum albumin (rhBSA) was loaded into LRNVs using sonication. CryoEM images of (A) empty, sonicated LRNVs and (B) rhBSA-loaded LRNVs shown as single particles in two fields of view. Resulting rhBSA-loaded LRNVs were measured for (C) size and (D) ζ-potential. (E–I) Uptake and internal trafficking of free rhBSA and rhbSA-LRNV into HUVECs were visualized with confocal microscopy. Representative images of rhBSA uptake and colocalization with (E) endosomes and (F) lysosomes at 1 and 6 h postincubation are shown. Red: free rhBSA or rhBSA-LRNVs, blue: nuclei, green: EEA1 (endosome) or LAMP1 (lysosome). Colocalization is indicated in yellow. Scale bar: 20 μm. (G) Images were quantified for total uptake using integrated density measurements. Particle colocalization in (H) endosome or (I) lysosome was quantified using Pearson’s coefficients. *n = 2* biological replicates, three fields of view per replicate were used for quantification. ***p < 0.001, ****p < 0.0001 using a two-way analysis of variance (ANOVA) with Bonferroni *post hoc* test.

Next, we carried out a proof-of-concept study to load exogenous cargo to explore the potential of LRNVs as drug nanocarriers. Tetramethylrhodamine-conjugated bovine serum albumin (rhBSA) was used as a model protein for cargo loading via sonication. Sonication has been previously applied as a cargo loading technique for EVs.^37^ In this method, 1 × 10^10^ LRNVs and 50 μg rhBSA were incubated together, and high frequency sound waves were applied to create micropores within the LRNV membrane and allow for the entry of the rhBSA molecules into the LRNV. Sonication was then stopped to allow the membrane to reform and close the pores, thus trapping rhBSA within the vesicle. Free rhBSA was removed using ultrafiltration and measured using UV spectroscopy to quantify loading efficiency. Sonication slightly decreased the LRNV concentration, though not significantly (*p* = 0.49, Figure S7A), but did not damage membrane proteins (Figure S7B). Membrane integrity was also not impacted, but sonication did lead to the formation of multilamellar vesicles (Figure 8A). General qualitative comparisons showed that the inner cores of the rhBSA-loaded LRNVs were more dense than unloaded LRNVs, potentially suggesting that rhBSA was incorporated inside the vesicle (Figure 8B). The size of the rhBSA-loaded LRNVs was almost unchanged compared to the empty LRNVs, indicating that loading of the rhBSA did not impact the overall particle size (Figure 8C). However, sonicated LRNVs, regardless of cargo loading, exhibited a slight increase in size compared to the extruded LRNVs (Figure 4A), potentially as a result of LRNV agglomeration due to sonication.^37,38^ The ζ-potential of empty LRNVs remained consistently around −20 mV, suggesting that sonication does not impact the overall surface charge. However, the ζ-potential of rhBSA-loaded LRNVs increased, though not significantly, compared to the empty LRNVs (Figure 8D). This may suggest that some rhBSA may have incorporated within or on the membrane rather than inside the vesicle, thus altering the overall surface charge. To assess the amount of rhBSA successfully loaded within the LRNVs, rhBSA in the ultrafiltration elution was measured using UV spectroscopy. Absorbance measurements were calculated against a rhBSA standard curve to obtain mass values of rhBSA within the elution. Unloaded LRNVs and rhBSA-only samples were used as background controls to eliminate any potential absorbances from the LRNV membrane proteins and to account for any rhBSA that was not completely removed during ultrafiltration. The average encapsulation efficiency was measured to be about 52.2% with 26.1 μg rhBSA loaded into 1 × 10^10^ particles.

Next, we investigated the uptake of the rhBSA when delivered by LRNVs. HUVECs were incubated with 5 × 10^8^ rhBSA-loaded LRNVs or an equivalent amount of free rhBSA (1.31 μg, based on average encapsulation efficiency) for 1 or 6 h. Cells were subsequently fixed and stained for the early endosome marker, EEA1, and the lysosome marker, LAMP1, to visualize rhBSA intracellular trafficking (Figure 8E,F). Both rhBSA and rhBSA-LRNV are uptaken by cells in a time-dependent manner with significantly more accumulation of the rhBSA-LRNV group at 6 h compared to 1 h (Figure 8G). At the 6 h timepoint, rhBSA-LRNVs had greater uptake compared to rhBSA alone, suggesting that LRNVs may facilitate faster uptake kinetics. However, the route of internalization and intracellular trafficking was observed to be similar between both groups. At 1 h, free rhBSA and rhBSA-LRNVs were found to partially colocalize with the endosomes (Pearson’s colocalization coefficient = 0.208 for free rhBSA and 0.213 for rhBSA-LRNV) and lysosomes (Pearson’s colocalization coefficient = 0.229 for free rhBSA and 0.251 for rhBSA-LRNV) (Figure 8H,I). Endosomal colocalization decreased at 6 h (Pearson’s colocalization coefficient = 0.183 for free rhBSA and 0.17 for rhBSA-LRNV), while lysosomal colocalization increased significantly (Pearson’s colocalization coefficient = 0.496 for free rhBSA and 0.443 for rhBSA-LRNV). These findings suggest that both free rhBSA and rhBSA-LRNV are internalized through an endocytic pathway as they initially colocalize within the early endosome before being eventually trafficked to the lysosome.

## 3 Discussion

EVs have emerged as powerful therapeutics to potentiate tissue regeneration due to their recognized roles as mediators of intracellular communication and transporters of vital proteins, lipids, and nucleic acids. However, the large-scale translation of EV therapeutics into clinical use faces major challenges due to low yields, complex isolation procedures, and innate functional heterogeneity. Here, we sought to engineer bioinspired nanovesicles by taking advantage of the known biogenesis pathways of EVs, namely that of exosomes. Exosomes are unique among other types of EVs in that they largely originate from the organized invagination of the lipid raft subdomains before being sorted into the endosomal compartments within the cell and ultimately secreted through the plasma membrane.^14,24^ As a result of this invagination, many of the lipids and proteins in the lipid rafts are thought to be transferred to the exosomal membrane. Due to this biogenesis pathway, we hypothesized that engineered nanovesicles synthesized from PMSC-derived lipid rafts would display similar structural features to native PMSC exosome membranes. We further sought to show that similarities in membrane composition could allow LRNVs to promote neuroregeneration and angiogenesis through membrane-bound molecular signaling.

Lipid rafts were isolated using a gradient ultracentrifugation technique where detergent-resistant membranes were collected and characterized as lipid rafts. These lipid raft isolates were strongly positive for the expression of caveolin-1, a well-known lipid raft marker, and general EV markers, indicating the successful isolation of lipid rafts that retained EV-like characteristics. LC/MS proteomic analysis revealed the presence of some contamination of cytosolic proteins coisolated with the lipid raft membranes, indicating that an additional gradient ultracentrifugation step may be useful to further purify isolates from any internal organelle protein contamination. We first validated the use of lipid rafts as biomaterials for LRNVs using bioinformatic analysis. Lipidomic analysis compared the lipidome profile of the lipid rafts and EVs isolated from three PMSC cell lines. Of the 619 lipid ions identified in both the lipid rafts and EV samples, 207 lipids were differentially expressed, indicating a 66.6% similarity between lipid rafts and EVs. The disparity in lipid expression was mainly due to variation in the percentages of glycerophospholipids and sphingolipids present in the two groups. Namely, lipid rafts have a higher expression of phosphatidylcholine but a lower expression of sphingomyelin compared to EVs. EVs are known to be enriched in sphingomyelin compared to source cells, possibly due to the vital role that sphingomyelin plays in supporting the formation of the intravesicular membrane of the multivesicular bodies (MVB) from which exosomes originate.^39,40^ Since the lipid raft isolation focused primarily on the collection of detergent-resistant cell membranes, this may have excluded lipids from the MVBs and thus account for the differentially expressed lipids. Unlike sphingomyelin, the expression of phosphatidylcholine is not universally enriched in exosomes and is instead dependent on the source cell from which they are derived.^41^ In previous studies and in our current work, EVs derived from MSCs were found to have lower phosphatidylcholine content compared to source cells.^42^ This may be due to the role phosphatidylcholine plays as a structural lipid constituent of the outer leaflet of the cell plasma membrane bilayer, including in MSCs.^43^ Thus, the higher percentage of phosphatidylcholine in the lipid rafts suggests more cell-like features than exosome-like characteristics. Furthermore, when comparing the overall lipidomes of whole cells, lipid rafts, and EVs, lipid rafts shared characteristics with both cells and EVs as the relative expression of most lipid classes within the lipid rafts most commonly fell between EVs and cell lysate expressions. This indicates that the lipid rafts retain cell-like features but also still display specific enrichments of certain lipid classes that are more reminiscent of EV lipidomes. Furthermore, proteomic comparisons between EV and lipid rafts show that the proteomes of the two are about 81.8% similar. This suggests that the proteomic profile of lipid rafts is very similar to PMSC EVs and that they may share many of the membrane protein-dependent biological functions of native PMSC EVs as shown in our previous work.^19^ The remaining differences in protein expression may be attributed to EV protein cargo missing from the lipid rafts or other extraneous cytosolic proteins that may have been coisolated with lipid rafts. Moreover, GO analysis of the common proteins found in all three donor lipid raft isolates revealed protein involvement in many signaling-dependent functions, such as RNA processing and biomolecular binding. KEGG pathway analysis identified that the lipid raft proteins additionally play key roles in regulating metabolic pathways. Furthermore, lipid raft isolates from different donor cell lines also exhibited similar lipidomic and proteomic profiles, thus highlighting the overall homogeneity of the lipid raft biomaterials for downstream functions. Combined, lipidomic and proteomic analyses revealed that lipid rafts have comparable lipidomic and proteomic profiles to EV membranes and demonstrate significant biological potential.

We further pursued the use of lipid rafts as a functional biomaterial by synthesizing lipid raft-derived nanovesicles, or LRNVs. LRNVs were generated by extrusion as this method has been previously used for synthetic liposomes and for shaping cellular membranes into homogeneous vesicles.^44−47^ LRNVs exhibited size, morphology, and stability profiles that were very similar to native EVs. LRNVs were able to be consistently synthesized at a size of about 120 nm and remained hydrodynamically stable over two weeks but decreased in concentration over time. However, the ζ-potential was found to be more variable over time. In particular, there was a noticeable trend of an increase in the ζ-potential and then a subsequent decrease over time. The increase in the ζ-potential can potentially be attributed to the slow degradation of the particles and the disintegration or shedding of surface molecules, which would remove the previously anionic surface proteins to reveal slightly more cationic molecules comprising the rest of the LRNV particle. The eventual return to a more negative plateau toward the end of the study period could reflect the surface charges of the remainder of the LRNVs that remained mostly whole and had yet to fragment or lose the outer anionic surface proteins. These trends are supported by other reported studies where EV storage resulted in a reduced concentration^48−50^ and increased ζ-potential^51^ over time. Size changes were more variable with one study reporting small increases of 10 nm in diameter,^51^ while others reported no observable size change.^49,50,52^ These studies hypothesized membrane protein degradation to be the main mechanism of loss of EV yield, which could have similar implications for the colloidal stability of the LRNVs in this study. Future stability studies could investigate the improvement of vesicle stability with the addition of protease inhibitors^48^ or buffers and additives such as trehalose^53^ or HEPES,^52^ or long-term storage at −80 °C.^49^

LRNVs were also synthesized at a relatively high amount, which addresses the previous challenge of limited yield faced by native EVs. These LRNVs also retained tetraspanin markers following synthesis, suggesting that the extrusion-based methodology did not overtly impact the structure or spatial orientation of the proteins on the membrane. Furthermore, both LRNV and EV tetraspanin expressions were quite similar, further highlighting the resemblance of LRNVs to EV membranes. Protein and lipid functions were also found to be conserved as we found that LRNVs retained the signaling properties of lipid rafts. Using galectin-1 and β-catenin as representative proteins, we confirmed that vital signaling proteins colocalized within the lipid rafts, further supporting the potential of LRNVs as important mediators of signal transduction. This was further confirmed by the ability of the LRNVs to upregulate the Akt pathway, a key signaling pathway for both neurogenesis and angiogenesis. Downstream validation of these functional properties was conducted with *in vitro* analyses of neuroregeneration and angiogenesis. We found that LRNVs were able to stimulate neurogenesis and promote neural recovery following apoptotic injury. LRNVs also demonstrated some pro-angiogenic properties with the ability to significantly improve tubulogenesis and endothelial cell migration but not cell proliferation.

LRNVs, though biologically functional, can be engineered to have greater therapeutic efficacy by loading bioactive cargo. We performed a proof-of-concept protein loading with rhBSA. Successful entrapment of rhBSA within LRNVs highlights the potential of these nanovesicles as a drug delivery system. Uptake and trafficking studies show that the LRNVs can facilitate a faster and increased uptake of rhBSA at least partially through an endocytosis-dependent mechanism. This could be potentially attributed to LRNV surface protein interactions with target cell membrane proteins that may activate endocytosis pathways.^54^ Free rhBSA, on the other hand, has less of an interactive tactic effect, and thus, these findings suggest the need for packaging of these cargo molecules within LRNVs for improved uptake. However, there remains a concern of cargo adhesion on the LRNV membrane, which may affect stability of the vesicle. For example, we suspect that some of the rhBSA may have adsorbed onto the LRNV membrane surface as suggested by the increase in the ζ-potential. To overcome this, PEGylated lipids, which increases the hydration of the outer membrane layer, may be introduced to the LRNV membrane to prevent protein opsonization.^55,56^ Otherwise, zwitterionic lipids, which contain both positive and negative charges, can also be incorporated to prevent nonspecific protein binding to the nanovesicle surface.^57^

In this study, we show for the first time that lipid rafts can be used as a bioactive biomaterial for nanovesicle synthesis. Resulting LRNVs retained many physical and biological characteristics that are reminiscent of native exosomes and, as such, offer an alternative type of biological nanovesicle that can be used as nanotherapeutics. Unlike native EVs, LRNVs can be synthesized in a more facile method and at a much higher yield. LRNVs also present as a novel technology that can be readily optimized for targeted applications. In this current application, we sought to synthesize PMSC-derived LRNVs due to our previous observations that PMSCs and PMSC-derived EVs have significant pro-neurogenic and angiogenic properties. LRNVs can easily be synthesized from other cell types or primed cells for more disease-specific properties. Additionally, the ease of loading bioactive cargo allows for greater opportunity to synthesize highly functional and tailored therapeutics that can be manufactured on a large scale.

Nevertheless, further characterization and optimization are required to fully understand the clinical implications of LRNVs. This study did not precisely conduct single vesicle analysis to characterize the effects of extrusion and sonication on lipidomic and proteomic composition of the LRNVs. Though we still observed significant biological properties, it must be more thoroughly investigated to determine if these methodologies have altered some protein or lipid functions. Additional methodologies for cargo loading must also be studied to validate optimal parameters for maximal encapsulation efficiency and particle stability. Furthermore, assessment of cell targeting behavior *in vitro* and pharmacokinetic parameters *in vivo* is also required to stringently assess the long-term safety of the LRNVs and any cargo-loaded LRNVs.

Overall, LRNVs offer a new and exciting alternative to EV therapies as biological nanovesicles that can regulate a variety of biological functions through membrane-mediated signaling. LRNVs present a versatile platform with high potential for a wide range of prospective applications in regenerative medicine and tissue engineering.

## 4 Conclusions

In this study, we show that lipid rafts can be considered a bioactive biomaterial from which nanovesicles can be synthesized. Though not a perfect replica of native exosome or EV membranes, lipid rafts and the subsequent LRNVs nevertheless offer a novel alternative as biological nanovesicles that can be synthesized consistently and on a large scale. We demonstrate that PMSC-derived lipid rafts and LRNVs possess promising therapeutic functions that can be applied for both neuroregenerative and angiogenic applications. The biological efficacy of the bioinspired LRNVs sheds light on the innate regenerative properties of lipid rafts and suggests that such biological properties can be conserved even as engineered nanovesicles. Ultimately, LRNVs can be leveraged as a platform technology that may be readily modified to target specific diseases and disorders.

## 5 Experimental Methods

### 5.1 Cell Culture

PMSC cell banks used in this study were isolated from chorionic villus tissue from deidentified, discarded second trimester human placentas and characterized in our previous study.^58^ PMSCs were expanded in a T150 tissue culture-treated flask with D5 media containing Dulbecco’s Modified Eagle’s Medium (DMEM, HyClone) with high glucose, 5% fetal bovine serum (FBS, Atlanta Biologicals), 20 ng/mL recombinant human basic fibroblast growth factor (bFGF, R&D Systems), 20 ng/mL epithelial growth factor (EGF, R&D Systems), 100 U/mL penicillin and 100 μg/mL streptomycin at 37 °C, and 5% CO_2_ until they reached 90% confluence. Cells were used between passages P3 and P5 for all experiments. Human umbilical vein endothelial cells (HUVECs, Lonza) were expanded in EGM-MV2 media (PromoCell) and were used between P3 and P5 for all experiments. SH-SY5Y cells (ATCC) were cultured in DMEM high glucose containing 5% FBS, 100 U/mL penicillin and 100 μg/mL streptomycin (termed here as S-5 media) at 37 °C, and 5% CO_2_ and used up to 10 passages.

### 5.2 Isolation and Characterization of Lipid Rafts and EVs

#### 5.2.1 Lipid Raft Isolation

Flasks with 90–95% confluent PMSCs were gently scraped using a cell scraper and collected on ice. Cells were centrifuged at 500*g* for 10 min, 4 °C (Sorvall). The resulting cell pellets were pooled and washed with 20 mL of fresh ice-cold phosphate buffered saline (PBS, Gibco) and centrifuged at 500*g* for 10 min. The pellet was washed two more times with ice-cold PBS. After the final wash, the cell pellet was resuspended in lysis buffer (50 mM MES, 150 mM NaCl, 0.5% Triton-X-100, and 2% v/v protease inhibitor cocktail (Sigma-Aldrich)), homogenized with a Dounce homogenizer with 50 passes, and incubated on ice for 1 h. Cell lysate was mixed with OptiPrep solution (Sigma-Aldrich) to create a 35% layer and added to the bottom of a prechilled polypropylene ultracentrifuge tube (#343778, Beckman Coulter). Step OptiPrep gradient layers of 30, 25, 20, and 0% concentrations were created by mixing Optiprep solution with MBS buffer (50 mM MES, 150 mM NaCl) and sequentially added. All volumes are given in the Supporting Information (Tables S1 and S2). Care was taken to prevent the mixing of the gradients. The gradients were ultracentrifuged (SW60 or TLS-55 rotor, Optima TLX, Beckman Coulter) at 250,000*g* at 4 °C for 2.5 h (TLS-55) or 4 h (SW60). The volumes within the 20–25–30% gradient fractions were pooled and centrifuged at 250,000*g* for 40 min. The supernatant was discarded, and the lipid raft pellet was resuspended in 1 mL of PBS. The samples were aliquoted and stored at −80 °C until further use.

#### 5.2.2 Dot Plot Characterization of Lipid Rafts

The dot blot apparatus (Bio-Rad) was operated according to the manufacturer’s instruction. The nitrocellulose membrane was rinsed with Tris-buffered saline (TBST) containing 20 mM Tris-HCl (pH 7.4), 150 mM NaCl, and 0.5% Tween-20. Different gradient fractions collected during lipid raft isolation were loaded and allowed to incubate for 30 min at room temperature. The wells were blocked with 1% bovine serum albumin (BSA) in TBST for 1 h at room temperature. The membrane was probed with caveolin-1 (#3238, Cell Signaling Technology, 1:1000 dilution), GRASP55 (#PA5-57210, Thermo Fisher, 1:500), and HSP60 (#1D11BD8, Abcam, 1:1000) in 1% BSA and incubated for 40 min. After washing the membrane three times with TBST, wells were incubated with anti-Rabbit HRP secondary antibody at a 1:2500 dilution incubated for 40 min at room temperature. The membrane was washed three times with TBST and developed using ChemiDoc XRS+ System (Bio-Rad) with Image Lab software.

#### 5.2.3 SDS-PAGE Protein Profile Comparison

Five micrograms each of cell lysates and lipid rafts isolated from the same cell line were resolved on a 4–15% NuPAGE Bis-Tris gel (Thermo Fisher) at constant 150 V for 90 min. Proteins were stained with Imperial Protein Stain (Thermo Fisher) for 1 h. Stained gels were visualized using a ChemiDoc XRS+ System (Bio-Rad).

#### 5.2.4 EV Isolation

EVs were isolated as described previously.^19^ Briefly, PMSCs at P5 were seeded at 20,000 cells/cm^2^ in five-layer flasks and cultured in EV-depleted D5 media for 48 h at 37 °C and 5% CO_2_. Conditioned medium was collected and filtered through a 0.2 μm filter to remove cells and cell debris. Conditioned medium was then centrifuged at 2000*g* for 30 min. The supernatant was transferred to thickwall polypropylene tubes (355462, Beckman Coulter) and centrifuged at 8836*g* for 30 min (SW-28 rotor, Optima XL-100, Beckman Coulter) to eliminate larger EVs. The pellet was discarded, and the supernatant was transferred to fresh tubes and centrifuged at 112,700*g* for 90 min. The resulting EV pellet was resuspended with PBS and centrifuged once more at 112,700*g* for 90 min. The pellet was resuspended in 50 μL of triple-filtered PBS (Gibco) per five-layer flask. EVs were aliquoted and stored at −80 °C until use.

#### 5.2.5 Western Blot Characterization of Lipid Rafts and EVs

Samples were loaded onto 4–15% NuPAGE Bis-Tris gel (Thermo Fisher Scientific) and run at a constant 150 V for 90 min in 2-(*N*-morpholino) ethanesulfonic acid buffer (Thermo Fisher Scientific). After completion of the run, the proteins were transferred to a nitrocellulose membrane at a constant voltage of 100 V for 45 min in transfer buffer (25 mM Tris-base, 200 mM glycine and 20% methanol). The membrane was probed with 1:500 dilution of primary antibodies ALIX (#SAB4200476, Sigma-Aldrich), tumor susceptibility gene 101 (TSG101) (#T5701, Millipore Sigma), CD9 (Millipore Sigma), CD63 (Thermo Fisher Scientific), CD81 (#5A6, EMD Millipore), and 1:1000 dilution of caveolin-1 and HSP60 diluted in 5% nonfat dry milk in TBST overnight at 4 °C. The membrane was probed with appropriate secondary antibodies and developed using ChemiDoc XRS+ System with Image Lab software (Bio-Rad).

### 5.3 Lipidomic Characterization of Lipid Rafts and EVs

Lipidome profiling of EVs and lipid rafts were performed by BGI-Americas (San Jose, CA). Lipids were extracted with a 3:1 (v/v) solution of dichloromethane/methanol and small steel balls. Lipid extracts were lyophilized and reconstituted in 2:1:1 (v/v) solution of isopropanol/acetonitrile/water. Liquid chromatography–tandem mass spectroscopy (LC-MS/MS) analysis was used for lipid separation and detection. Lipid separation was performed with ultrahigh performance liquid phase chromatography (Waters 2D UPLC, Waters) with a CSH C18 column. The injection volume was 5 μL, and the flow rate was 0.35 mL/min. MS analysis was performed on a Q-Exactive mass spectrometer (Thermo Fisher Scientific). MS data were acquired by selecting the top three ions according to the precursor ion intensity from the survey scan (200–2000 *m*/*z*). Lipids were identified and quantified using LipidSearch v.4.1 (Thermo Fisher Scientific) using the following parameters: quality deviation of precursor ions: 5 ppm, quality deviation of product ions: 5 ppm, product ion threshold: 5.0%, peak area and peak extraction tolerance: 5 ppm. Quality control (QC) samples were used for data preprocessing. Lipid molecules that were missing more than 50% of the QC samples or had a coefficient of variation of a relative peak area greater than 30% in the QC samples were deleted. Missing values in the MS metabolomics dataset were filled using a KNN algorithm, and local polynomial regression fitting signal correction for real sample signals based on the QC sample (QC-RLSC) was used for data correction.

Differential expression of lipids between groups was screened using multivariate analysis and univariate statistical analysis. Data were normalized to total lipid intensities detected and log 2 transformed before all analysis. Principal component analysis (PCA) was performed using the *pca* function in Matlab v.R2019B to observe the distribution and separation between Pareto-scaled datasets for the cell lysate, EV, and lipid raft groups. Only lipids found in all groups were compared. Fold change analysis (FC analysis), followed by Student’s *t*-test statistical analysis, was conducted in RStudio v.1.4.1106 to identify differentially expressed lipid molecules between lipid rafts and EVs.

### 5.4 Proteomic Characterization of Lipid Rafts

Proteomic profiling was performed at BGI-Americas (San Jose, CA). Samples were denatured, digested, and analyzed by tandem mass spectrometry (LC-MS/MS) using the Q-Exactive HF-X mass spectrometer (Thermo Fisher Scientific). MS acquisitions were searched against the most updated Uniprot Homo sapiens database with Sequest analysis workflow to identify proteins in the samples. Gene ontology searches were performed using FunRich v.3.1.3, and KEGG pathway analysis was performed with Database for Annotation, Visualization, and Integrated Discovery (DAVID) v.6.7.^59−63^

### 5.5 LRNV Synthesis and Characterization

#### 5.5.1 LRNV Synthesis

The lipid raft pellet was resuspended in the 1× PBS and extruded 15 times using the mini-extruder (Avanti Polar Lipids) with polycarbonate filters of 200 nm pore size. Following synthesis, LRNVs were stored at 4 °C in water until use.

#### 5.5.2 Size, Concentration, and ζ-Potential

The size, concentration, and ζ-potential of the LRNVs were measured with nanoparticle tracking analysis (NTA) using the ZetaView (Particle Metrix). Samples were prediluted to an optimal concentration to allow for about 150 particles/frames (ZetaView v.8.05.12). For each measurement, 11 positions were scanned for two cycles using the following parameters: camera sensitivity: 92, shutter: 150, frame rate: 30, and cell temperature: 25 °C. Particle stability studies were conducted by monitoring changes in the size, concentration, and ζ-potential in water at 4 °C or in PBS at 37 °C over 15 days.

#### 5.5.3 Morphology

Cryogenic electron microscopy (cryo-EM) was used to visualize the LRNV morphology. Carbon EM grids (Ted Pella Inc.) were glow-discharged at 30 mA, 30 s (Pelco Auto Sputter Coater SC-7, Ted Pella Inc.). First, 4 μL of 1 × 10^11^ LRNV/mL solution was incubated on the carbon side of the EM grid, blotted for 5 s, and then plunge-frozen into a precooled vat of liquid ethane with Vitrobot Mark MkIII (FEI). Vitrified samples were imaged with the Glacios cryo-transmission electron microscope equipped with a K3 direct electron detector and acquired with SerialEM software (D.Mastronarde, Boulder Lab).

#### 5.5.4 ExoView Tetraspanin Detection

ExoView kits were used per manufacturer’s protocol (NanoView Biosciences). Chips were prescanned before use. LRNVs were diluted to a concentration between 1 × 10^8^ and 1 × 10^9^ particles/mL in the provided incubation solution and incubated on the chip overnight at room temperature. Following incubation, the chip was washed three times with incubation buffer and fluorescently labeled primary antibodies were added (1:500 dilution in blocking solution). The following antibodies were used: CF488-anti-CD9 (clone: HI9a), CF647-anti-CD63 (clone: H5C6), and CF555-anti-CD81 (clone: JS81). Chips were incubated with the antibodies for 1 h at room temperature while being shaken at 500 rpm. After incubation, chips were washed three times with incubation buffer, once in deionized (DI) water, and carefully dried on an absorbent paper. Once fully dried, the chips were scanned using the ExoView R100 (Nanoview Biosciences) for data acquisition.

#### 5.5.5 LRNV Uptake

HUVECs were grown to 90% confluence in 48-well tissue culture-treated plates. LRNVs were labeled with DiI or DiD dye (Thermo Fisher Scientific). Dyes were added to LRNVs at a final concentration of 1.67 μM and incubated at 37 °C for 10 min. Excess DiI was removed using Nanosep centrifugal ultrafiltration devices with Omega membranes (100 kDa MWCO, Pall OD100C34). To control for excess DiI micelles, the same procedure was conducted with the DiI dye alone with the LRNV sample volume replaced with 1× PBS instead. 5 × 10^8^ LRNV particles were added to each well and incubated for 4, 6, or 24 h. At the end of the timepoint, media was aspirated and replaced with fresh media supplemented with 20 μg/mL Hoechst 33342 (Thermo Fisher Scientific #62249) and imaged using a Carl Zeiss Axio Observer D1 inverted microscope. Particle uptake was assessed semiquantitatively using ImageJ. Before analysis, intensities of the control-DiI group were subtracted from the LRNV group at each timepoint to account for background. For representative images, cells were plated in eight-well chamber slides (Labtek), fixed with formalin for 20 min at room temperature (RT), and stained with DAPI before being mounted and imaged using a Nikon C2 scanning laser microscope.

### 5.6 Cell Signaling Characterization

#### 5.6.1 PMSC Immunostaining

PMSCs were fixed in 10% formalin for 20 min, RT and blocked with 3% BSA for 30 min. Cells were incubated with anti-caveolin-1 (1:200, #7C8, Santa Cruz Biotechnology), and anti-galectin-1 (1:200, #D608T, Cell Signaling Technologies) or anti-β-catenin (1:200, #MAB2081, R&D Systems) overnight at 4 °C. AlexaFlour647-conjugated anti-mouse and AlexaFlour555-conjugated anti-rabbit secondary antibodies were added at a 1:1000 dilution for 1 h, RT. 4′,6-Diamidino-2-phenylindole (DAPI) was added to visualize the cell nuclei. Nonspecific mouse and rabbit IgG controls were performed.

#### 5.6.2 Co-immunoprecipitation

Equal protein amounts of lipid rafts were incubated with 1 μg of anti-caveolin-1 antibody (#7C8) or nonspecific mouse-IgG (Santa Cruz Biotechnology) as negative control for 16 h at 4 °C. Protein A/G-Sepharose beads (Santa Cruz Biotechnology) were added and incubated for 1 h at 4 °C. Beads were precipitated by centrifugation and washed four times with PBS. Beads were boiled in SDS sample buffer for 10 min at 70 °C before being resolved by Western blotting as described above. The membrane was probed with anti-β-catenin (1:500), anti-galectin-1 (1:1000), and anti-caveolin-1 (#3238, 1:1000) antibodies.

#### 5.6.3 HUVEC Akt Signaling

HUVECs were grown to 80% confluence in six-well tissue culture-treated plates in EGM-MV2. Medium was then changed to 1% EGM-MV2, 1 × 10^9^ LRNV particles/mL were added, and then incubated for 48 h. Following incubation, cells were lysed in RIPA lysis buffer (Thermo Fisher Scientific) with 1× protease inhibitors cocktail (Millipore Sigma) and 1× phosphatase inhibitors (Santa Cruz Biotech). The protein concentration of the cell lysate was quantified using a Bicinchoninic Acid Assay Kit (Thermo Fisher Scientific) per manufacturer’s protocol. Akt activity was assessed using Western blotting. Fifteen micrograms of protein was loaded onto 4–12% Criterion Bis-Tris protein gels (Bio-Rad) run at a constant 150 V using 3-(*N*-morpholino) propanesulfonic acid buffer (Thermo Fisher Scientific). Proteins were then transferred onto a nitrocellulose membrane and probed with 1:1000 dilutions of Akt (#9272, Cell Signaling Technologies) and GAPDH (#6C5, Santa Cruz Biotech) antibodies.

### 5.7 Neurogenerative Properties

#### 5.7.1 Neurogenesis Assay

SH-SY5Y cells were seeded in 48-well plates at a density of 30,000 cells/well and allowed to adhere overnight. Medium was aspirated and 200 μL of fresh medium supplemented with 1 × 10^9^ LRNV particles/mL was added and incubated for 48 h. Cells were washed with PBS and stained for 2 min using 2 μM calcein AM (Thermo Fisher Scientific). Cells were imaged using a Carl Zeiss Axio Observer D1 inverted microscope, and total segment length and branching points were quantified using WimNeuron Image Analysis (Onimagin Technologies).

#### 5.7.2 Neuron Proliferation

The effect of LRNVs on SH-SY5Y cells was assessed using an MTS assay. SH-SY5Y cells were seeded at 10,000 cells/well in 96-well tissue culture-treated plates and cultured in S-5 medium for 24 h. Particles were added at a concentration of 1 × 10^9^ particles/mL and incubated for 48 h at 37 °C and 5% CO_2_. Cell proliferation was assessed using a CellTiter 96 AQueous One Solution Cell Proliferation Assay (MTS, Promega) according to the manufacturer’s instructions.

#### 5.7.3 Neuroprotection Assay

The neuroprotective ability of the LRNVs was investigated using a neuroprotection model established in our previously published study.^26^ Briefly, 100,000 SH-SY5Y cells/cm^2^ were seeded on 48-well plates and cultured for 24 h. Apoptosis was induced by treating the cells with 0.5 μM staurosporine (Cell Signaling Technology) for 4 h. Cells were carefully washed with media once, and 200 μL of fresh media containing 1 × 10^9^ LRNV particles/mL was added. At 5 days posttreatment, the cells were washed with PBS and stained for 2 min using 2 μM calcein AM. Cells were imaged using a Carl Zeiss Axio Observer D1 inverted microscope to observe the changes in neuronal survival after induced apoptosis. Total circuitry length, total branching points, and total segment length were quantified using WimNeuron Image Analysis.

### 5.8 Angiogenic Properties

#### 5.8.1 HUVEC Tube Formation

HUVECs were seeded onto growth factor-reduced Matrigel-coated (Corning) 96-well plates at 10,000 cells/well and cultured in 100 μL of EBM medium with or without 1 × 10^9^ LRNV particles/mL and incubated at 37 °C and 5% CO_2_. Images were taken at 6 h posttreatment using a Carl Zeiss Axio Observer D1 inverted microscope. The number of nodes, number of branches, and vessel density (mesh area/total area) were quantified using the Angiogenesis Analyzer tool on ImageJ (v.1.52p, NIH).

#### 5.8.2 HUVEC Migration

HUVECs were grown to confluence in 24-well plates and serum-starved in EBM media for 16 h before the start of the assay. A pipette tip was used a make a straight, vertical scratch in the middle of the well. EBM (300 μL) with 1% BSA media with or without 1 × 10^9^ particles/mL LRNVs was added to the HUVECs and incubated at 37 °C and 5% CO_2_. Images were taken at 8 h posttreatment using a Carl Zeiss Axio Observer D1 inverted microscope. The wound area was quantified using the following formula
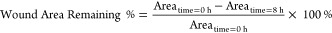


#### 5.8.3 HUVEC Proliferation

HUVECs were seeded at 2000 cells/cm^2^ on 96-well tissue culture-treated dishes and cultured in EGM-MV2 for 24 h. Cells were treated with or without 1 × 10^9^ particles/mL of LRNVs in 100 μL of EBM media with 1% BSA and incubated for 48 h at 37 °C and 5% CO_2_. Cell proliferation was assessed using the CellTiter 96 AQueous One Solution Cell Proliferation Assay (MTS, Promega) according to the manufacturer’s instructions.

### 5.9 Cargo Loading Proof of Concept

To confirm that exogenous molecules could be loaded into LRNVs successfully, tetramethylrhodamine-conjugated bovine serum albumin (rhBSA) was used as a model protein cargo. 1 × 10^10^ LRNV particles were mixed with 50 μg of rhBSA and sonicated in a water bath sonicator (Elmasonic P, Elma) under the following parameters: 37 kHz for 30 s and 1 min incubation on ice, followed by a second cycle of sonication at 37 kHz for 30 s and 1 min on ice. Samples were incubated on ice for an additional 15 min. The ratio of rhBSA to LRNVs was chosen based on preliminary experiments to establish the detection limits for the rhBSA.

The rhBSA loading was quantified using a Nanodrop 2000 spectrophotometer (Thermo Fisher Scientific). First, samples were filtered through Nanosep centrifugal ultrafiltration devices with Omega membranes (100 kDa MWCO, Pall Corporation, #OD100C34) to remove free rhBSA. Eluted free rhBSA was measured for tetramethylrhodamine absorbance, and the rhBSA concentration was calculated against a linear regression standard curve. To account for any potential absorbance from LRNV particles that have may been coeluted and any rhBSA that was not fully eluted, background control groups with LRNV-only and rhBSA-only were measured. Encapsulation efficiency (%) was measured as the following
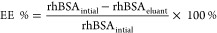


Uptake of rhBSA-LRNV particles was assessed in HUVECs as previously described. Briefly, 5 × 10^8^ particles of rhBSA-LRNV or equal amount of free rhBSA was added to the cells in an eight-well chamber slide (Labtek) and incubated for either 1 or 6 h. Following incubation, cells were fixed with formalin for 20 min, RT and washed three times with cold PBS. Cells were permeabilized with 0.5% Triton-X 100 and incubated with EEA1 (#C45B10, 1:200, Cell Signaling Technologies), LAMP1 (#D2D11, 1:200, Cell Signaling Technologies), and DAPI at 4 °C overnight to stain for the endosome, lysosomes, and nuclei, respectively. AlexaFlour647-conjugated anti-rabbit donkey secondary antibody (Thermo Fisher) was added at a 1:500 dilution for 1 h at RT. Slides were mounted and imaged using a confocal Nikon C2 laser scanning microscope. Maximal projections of z-stack images were used for data quantification. Colocalization analysis was performed using the *coloc2* function on FIJI/ImageJ (v1.52p, NIH) with PSF = 3 and 10 Costes randomizations. Particle uptake was semiquantified using integrated density values measured in ImageJ.

### 5.10 Statistical Analysis

Data are reported as means ± sd. Statistical analysis was performed using Prism v.8.4.3 software (Graphpad Software, La Jolla, CA). For comparisons between two groups, a *t*-test was performed. Comparisons between multiple groups were performed with ANOVA with a Bonferroni *post hoc* test. Differences were considered significant at a *p*-value < 0.05.

## Supporting Information Available

Tables for OptiPrep gradient preparation, characterization of PMSCs and EVs, lipid subclass comparisons between lipid rafts and EVs, size distribution of unextruded and extruded lipid rafts, cryoEM vesicles after 6 days in storage, background controls for uptake studies and ICC, and characterization of sonication effects on LRNV and membrane integrity. Proteomic dataset for PMSC and PMSC lipid rafts. Lipidomic dataset for PMSC, PMSC lipid rafts, and PMSC-EV (PDF)
Proteomic dataset for PMSC and PMSC lipid rafts (XLSX)
Lipidomic dataset for PMSC, PMSC lipid rafts, and PMSC-EV (XLSX)
