# Supplementary material for: Development and Characterization of Bioinspired Lipid Raft Nanovesicles for Therapeutic Applications
Source: ACS Appl Mater Interfaces. 2022 Nov 30;14(49):54458–77. doi: 10.1021/acsami.2c13868 (PMC9756296; doi:10.1021/acsami.2c13868)
Supplement: Supplementary file 1 — am2c13868_si_001.pdf [file am2c13868_si_001.pdf]

Supporting Information

Development and Characterization of Bioinspired Lipid Raft Nanovesicles for Therapeutic Applications

Lalithasri Ramasubramanian[1,2,3], Harsha Jyothi[1], Leora Goldbloom-Helzner[1,2,3], Brandon M. Light[1], Priyadarsini Kumar[1,2], Randy P. Carney[3], Diana L. Farmer[1,2], Aijun Wang[1,2,3]*

*Corresponding author: Aijun Wang, PhD, Email: aawang@ucdavis.edu

[1]Department of Surgery, School of Medicine, University of California-Davis, Sacramento, CA, 95817

[2]Institute for Pediatric Regenerative Medicine, Shriners Hospitals for Children, Sacramento, CA, 95817

[3]Department of Biomedical Engineering, University of California-Davis, Davis, CA, 95616

Table S1: The preparation of OptiPrep™ gradient using SW60 rotor (volumes given per tube)

**Gradient**	**OptiPrep™ percentage** (%)	**Cell lysate** (µL)	**MBS buffer** (µL)	**OptiPrep™ solution** (µL)	**Total volume** (µL)
1 (bottom)	35	378	0	522	900
2	30	—	500	500	1000
3	25	—	580	420	1000
4	20	—	650	350	1000
5 (top)	0	—	1000	—	1000

Table S2: The preparation of OptiPrep™ gradient using TLS55 rotor (volumes given per tube)

**Gradient**	**OptiPrep™ percentage** (%)	**Cell lysate** (µL)	**MBS buffer** (µL)	**OptiPrep™ solution** (µL)	**Total volume** (µL)
1 (bottom)	35	83.3	0	116.7	200
2	30	—	100	100	200
3	25	—	116.7	83.3	200
4	20	—	133.3	66.7	200
5 (top)	0	—	200	0	200

C

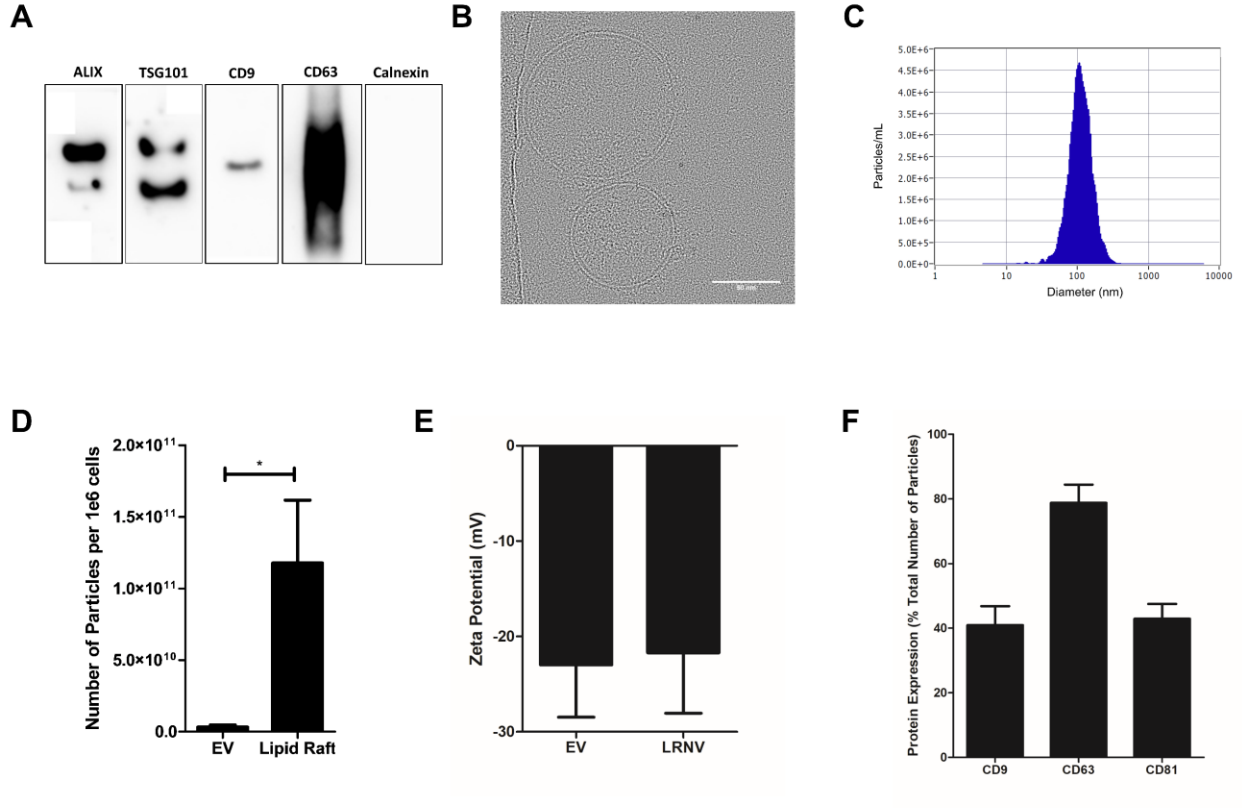

Figure S1. Characterization of PMSC EVs. (A) Representative Western blot of PMSC EV showing expression of accepted EV markers ALIX, TSG101, CD9, CD63 and negative expression on calnexin as the control. (B) Cryo-EM micrograph of PMSC EVs. Scale, 50 nm. (C) Size distribution of EV measured using NTA. (D) Particle yield of EV or lipid raft normalized to $1x10^6$ cells. (E) Zeta potential measurements of EV and LRNV. (F) ExoView analysis of CD9, CD63, and CD81 surface protein expression on PMSC EVs. *n*=3 biological replicates.

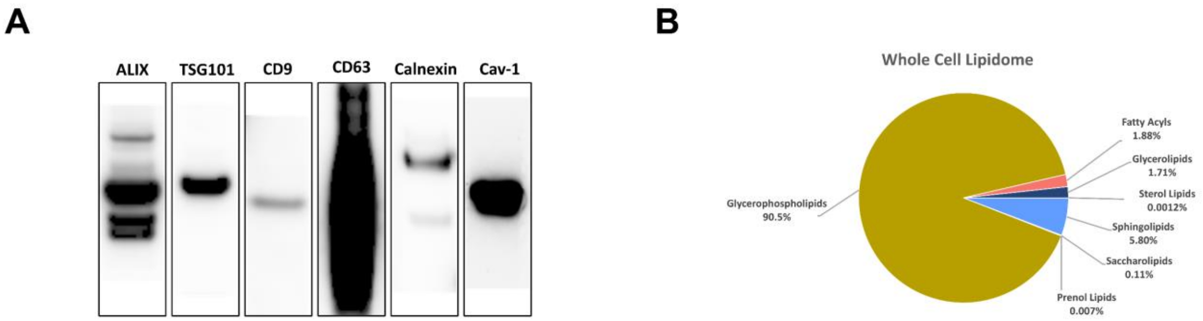

Figure S2. Characterization of PMSC whole cell. (A) Representative Western blot of PMSC cell lysate. (B) Lipidome profile of one PMSC cell line.

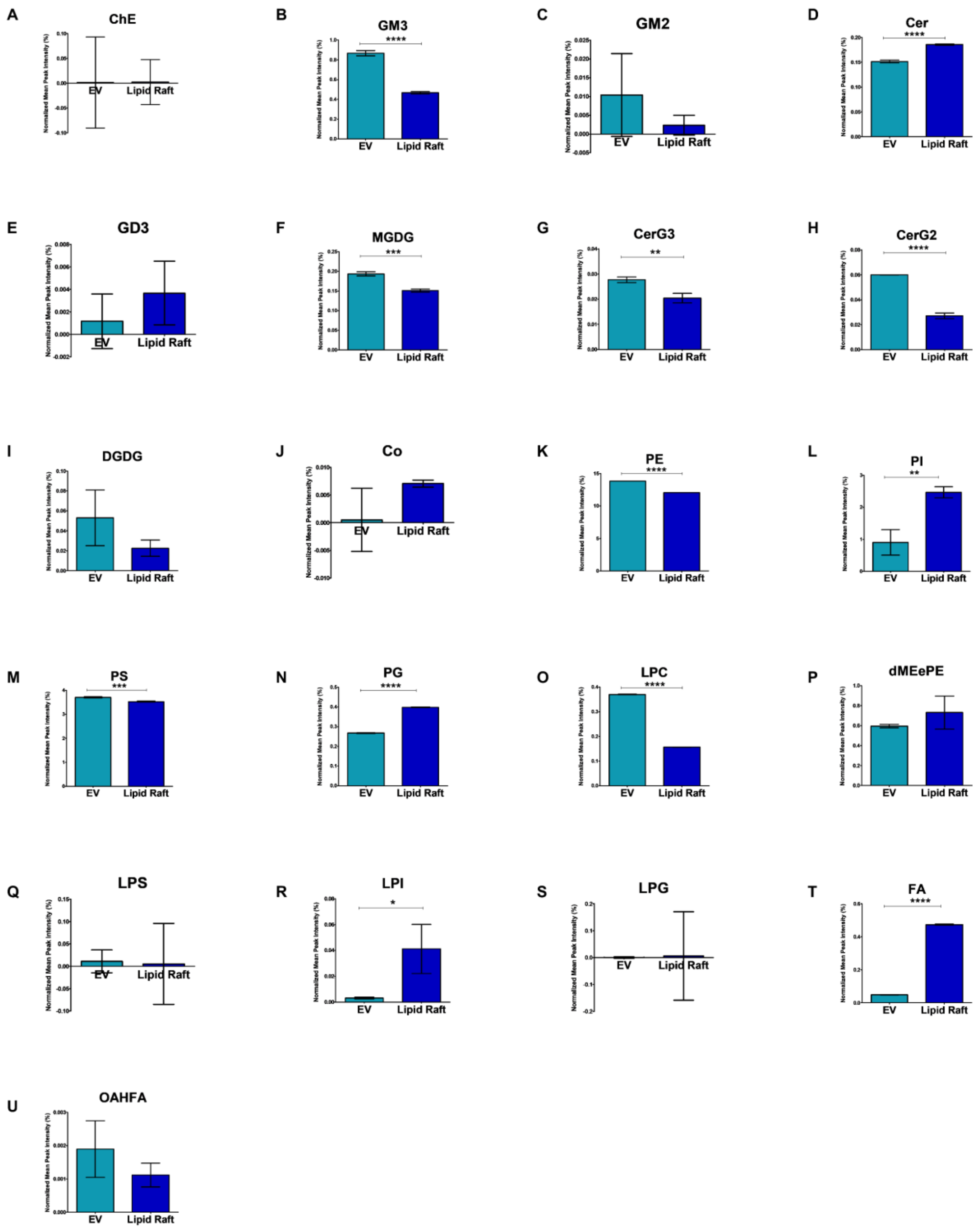

Figure S3. Quantitative analysis of relative amounts of different lipid subclasses detected in PMSC lipid rafts and EVs using LC-MS/MS. (A) ChE. (B) GM3, (C) GM2, (D) Cer, (E) MGDG, (F) CerG3, (G) CerG2, (H) DGDG, (I) Co, (J) PE, (K) PI, (L) PS, (M) PG, (N) LPC, (O) LPC, (P) dMEePE, (Q) LPS,

(R) LPI, (S) LPG, (T) FA, (U) OAHFA. *n*=3 cell lines. * $p<0.05$, ** $p<0.01$, *** $p<0.001$, **** $p<0.0001$ using a Student's t-test.

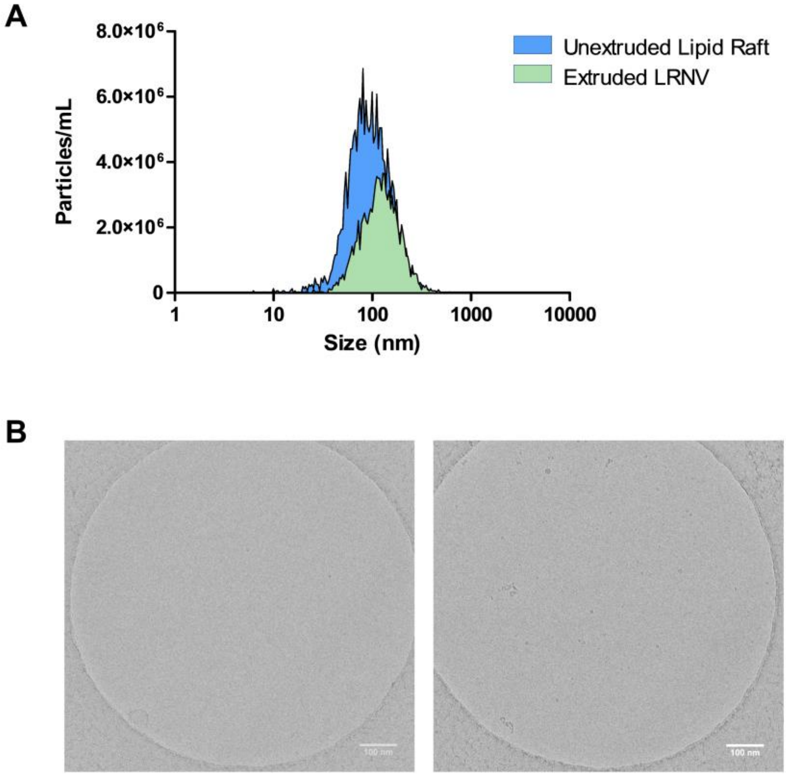

Figure S4. LRNV physical characteristics. (A) Size distribution comparison of unextruded lipid raft samples (blue) and extruded LRNV (green). Extrusion is seen to create LRNVs that have a larger mean size and more uniform size distribution compared to unextruded lipid raft isolates that self-assemble into more smaller and more heteregnously sized particles. (B) CryoEM imaging of LRNV after 6 days storage at 4°C.. Little to no LRNV particles were visualized in the sample. Two representative fields of view are shown.

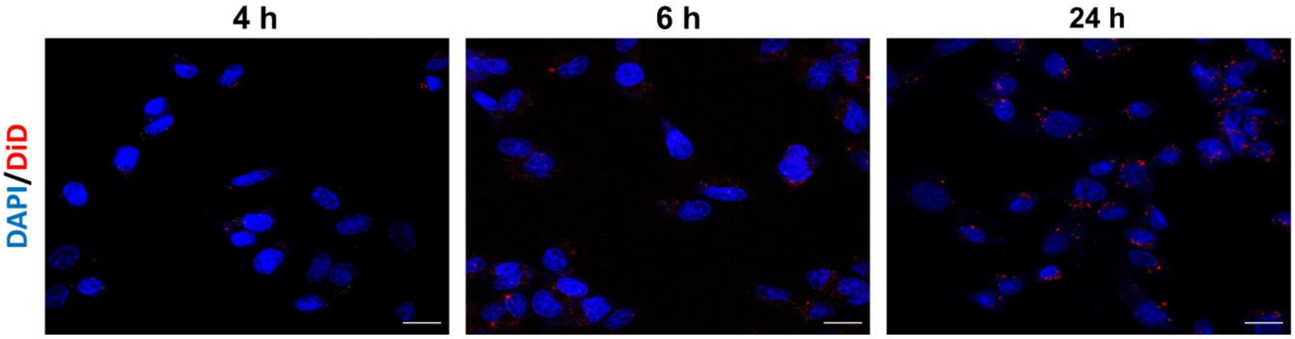

Figure S5. Free DiDbackground control for HUVEC uptake. As DiD dye can form nanometer-sized micelles, control DiD-only samples were prepared using the same methodology as DiI-LRNV samples. Equal volume of DiD-only suspension was added to HUVECs and incubated for 4, 6, and 24 h. DAPI

(blue) was added to visualize cell, and free DiD uptake into cells is seen in red. Minimal free DiD was visualized in the background. Scale, 20 µm.

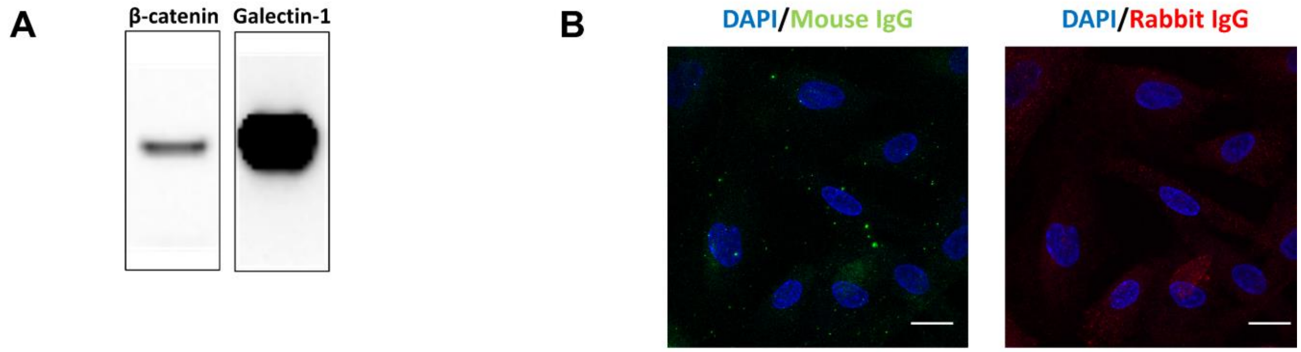

Figure S6. (A) Western blot analysis of PMSC EV for the positive expression of β-catenin and galectin-1. (B) Immunostaining of PMSCs with IgG controls for nonspecific mouse (green) and rabbit (red) IgG were performed to account for background staining during immunocytochemistry. Nuclei are visualized with DAPI (blue). Scale, 20 µm.

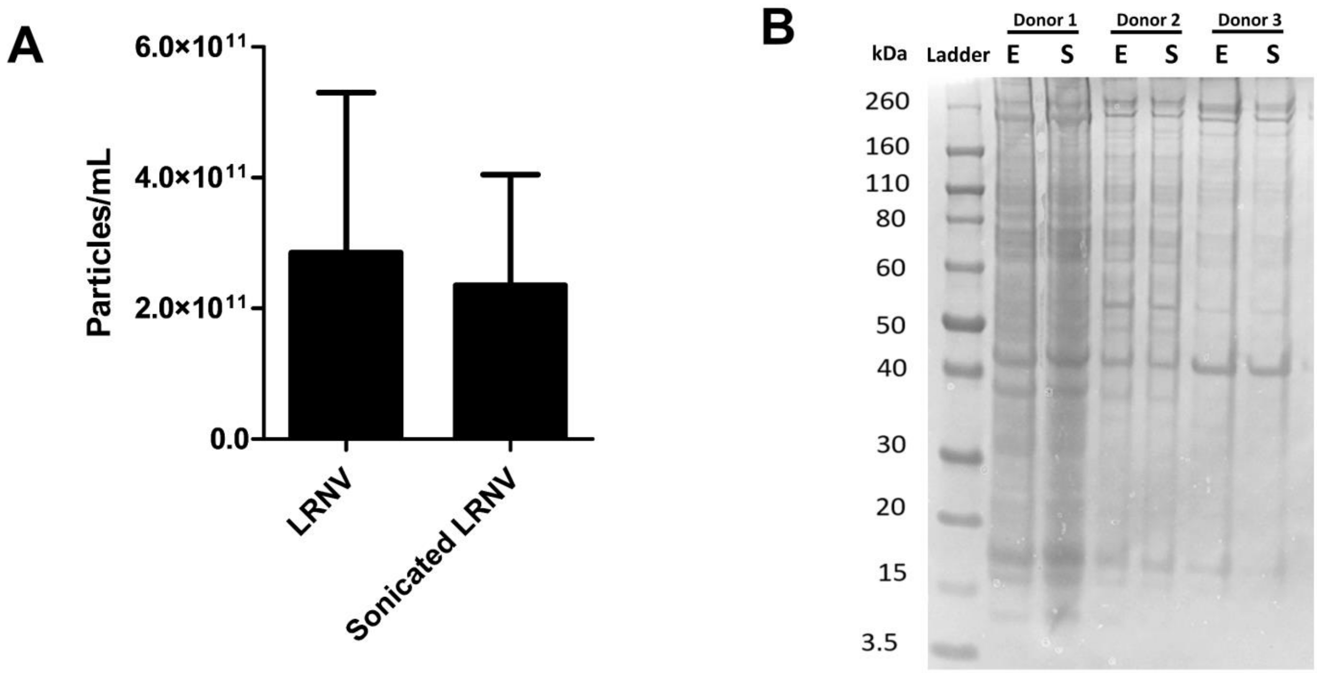

Figure S7. Effects of sonication on lipid rafts. (A) Concentration of LRNVs before and after sonication by NTA analysis. (B) SDS-PAGE of non-sonicated lipid rafts (*E*) and sonicated lipid rafts (*S*). Equal protein weights were loaded within each matched sample. Three cell lines are shown.
